# Tumor Necrosis Factor‐Alpha Inhibits the Replication of Patient‐Derived Archetype BK Polyomavirus While Activating Rearranged Strains

**DOI:** 10.1002/jmv.70210

**Published:** 2025-02-14

**Authors:** Lise Lauterbach‐Rivière, Lucia Thuringer, Pascal Feld, Lina Kathrin Toews, Jessica Schüssler, Jonas Klinz, Lars Gläser, Stefan Lohse, Anna Sternjakob, Gilles Gasparoni, Kathrin Kattler‐Lackes, Jörn Walter, Marcel A. Lauterbach, Sven Rahmann, Lars Möller, Michael Laue, Martin Janssen, Michael Stöckle, David Schmit, Danilo Fliser, Sigrun Smola

**Affiliations:** ^1^ Institute of Virology Saarland University Medical Center Homburg Germany; ^2^ Helmholtz Institute for Pharmaceutical Research Saarland (HIPS), Helmholtz Centre for Infection Research Saarland University Campus Saarbrücken Germany; ^3^ Department of Genetics Saarland University Saarbrücken Germany; ^4^ Molecular Imaging, Center for Integrative Physiology and Molecular Medicine Saarland University Homburg Germany; ^5^ Algorithmic Bioinformatics, Center for Bioinformatics Saar, Saarland Informatics Campus Saarland University Saarbrücken Germany; ^6^ Advanced Light and Electron Microscopy, Centre for Biological Threats and Special Pathogens, Robert Koch Institute Berlin Germany; ^7^ Department of Urology Saarland University Medical Center Homburg Germany; ^8^ Department of Nephrology Saarland University Medical Center Homburg Germany

**Keywords:** antiviral therapy, archetype, BK polyomavirus (BKPyV), drug discovery, IFN‐γ, non‐coding control region (NCCR), TNF‐α

## Abstract

To date, no drugs are approved for BK polyomavirus (BKPyV) reactivation, a major cause of nephropathy after kidney transplantation. Recently, tumor necrosis factor‐α (TNF‐α) blockade has been proposed as a promising therapy, however, the effect of TNF‐α on the clinically most common archetype (ww) BKPyV remained unclear. Assays in primary renal proximal tubule epithelial cells (RPTEC) allowed efficient replication only of BKPyV strains with rearranged (rr) non‐coding control regions (NCCR), which may develop at later disease stages, but not of ww‐BKPyV. Here, we optimized culture conditions allowing robust replication of patient‐derived ww‐BKPyV, while efficiently preserving their ww‐NCCR. TNF‐α promoted rr‐BKPyV replication, while the T_H_1 cytokine IFN‐γ suppressed it, also in the presence of TNF‐α. Surprisingly, TNF‐α alone was sufficient to suppress all ww‐BKPyV strains tested. Comprehensive analysis using siRNAs, and chimeric or mutated BKPyV‐strains revealed that the response to TNF‐α depends on the NCCR type, and that the NF‐κB p65 pathway but not the conserved NF‐κB binding site is essential for the TNF‐α‐induced enhancement of rr‐BKPyV replication. Our data suggest that in immunosuppressed patients with archetype‐dominated infections, TNF‐α blockade could interfere with natural TNF‐α‐mediated anti‐BKPyviral control, and this could be detrimental when IFN‐γ‐driven T_H_1 responses are impaired. Ongoing inflammation, however, could lead to the selection of rearrangements responding to NCCR‐activating pathways downstream of NF‐κB p65 signaling, that may overcome the initial TNF‐α‐mediated suppression. Our findings also highlight the importance of using clinically relevant BKPyV isolates for drug testing and discovery, for which this new assay paves the way.

## 1 Introduction

The BK polyomavirus (BKPyV) was first isolated in 1971 from the urine of a kidney transplant patient with the initials B.K. [1]. BKPyV is a member of the *Polyomaviridae* family and belongs to the *Betapolyomavirus* genus. BKPyV seroprevalence in adults is high and reaches more than 80% in individuals over 21 years of age [2]. Although asymptomatic in healthy individuals, BKPyV can reactivate in immunosuppressed individuals, particularly kidney or hematopoietic stem cell transplant recipients. Lytic infection in the transplanted kidney or the bladder urothelium may result in severe diseases, such as polyomavirus‐associated nephropathy (PVAN) or hemorrhagic cystitis [3]. PVAN is diagnosed in 1%–10% of all kidney transplant recipients and is associated with significant viremia and increased risk of graft loss [3, 4, 5]. Treatment of BKPyV‐associated disease remains a major challenge, since no anti‐BKPyV FDA‐approved drugs are available and reducing immunosuppression harbors the risk of graft rejection [6].

The cell‐mediated immune response is central in controlling BKPyV infection and PVAN development [7, 8], which raises interest in understanding the role of immune‐activating and pro‐inflammatory cytokines. In particular, interferon‐γ (IFN‐γ) and tumor necrosis factor‐α (TNF‐α) may synergize to induce a broad antiviral state [9].

IFN‐γ, a type II interferon, is produced by activated T helper 1 (T_H_1) and cytotoxic T lymphocytes as well as natural killer (NK) cells, and activates the Jak‐STAT pathway to induce IFN‐γ‐stimulated genes, including broad antiviral genes [10]. IFN‐γ inhibits the replication of BKPyV strains with rearranged non‐coding control regions (NCCR) in renal proximal tubule epithelial cells (RPTEC) [11]. TNF‐α is a pro‐inflammatory cytokine mainly produced by macrophages, but also T_H_1 lymphocytes, and NK cells. Upon receptor binding, TNF‐α can activate Nuclear Factor kappa B (NF‐κB) [12], other pro‐inflammatory pathways and secondary factors that induce pro‐inflammatory responses [13]. Increased levels of TNF‐α and its receptors TNFR1 and TNFR2 have been observed in the urine of PVAN patients [14]. Moreover, BKPyV upregulates both receptors in human collecting duct epithelial cells infected in vitro [15]. In contrast to IFN‐γ, TNF‐α has been shown to promote BKPyV replication in RPTEC through NF‐κB pathway activation [14]. Furthermore, binding of the NF‐κB subunit p65 to the NCCR of the rearranged Dunlop strain was shown to activate the early promoter [16]. Therefore, the initiation of clinical trials on TNF‐α blockade has recently been proposed to treat BKPyV infection [14].

To date, most studies on the BKPyV life cycle were performed with laboratory‐adapted strains, with “rearranged” (rr‐) NCCR, harboring insertions and/or deletions, whereas the major transmitted and reactivated viral forms in patients have an “archetype” (ww‐) NCCR [17, 18]. Rearrangements can alter the activity of the viral promoters and enhancers contained in the NCCR [19]. Although rearrangements can occur in vivo, they are only found in a minority (24%) of the patients with BKPyV viremia and in 50% of patients with PVAN [17]. Therefore, rr‐strains are not a prerequisite for PVAN but have been associated with higher early gene expression and replication rates [17, 18].

Suppressing BKPyV replication in patients at risk before rearranged and apparently more pathogenic strains emerge would be the ideal strategy [17]. However, the development of drugs against patient‐derived ww‐BKPyV has been hampered by the lack of a replication system for ww‐BKPyV in primary RPTEC, the target of BKPyV in PVAN. To date, ww‐BKPyV cannot be adequately propagated in RPTEC [20, 21], and viral reactivation is typically associated with NCCR rearrangements [22]. Similar issues were faced when using other cell types [23, 24, 25, 26]. Only in the HEK 293TT cell line, which ectopically overexpresses the large T antigen (TAg) of polyomavirus SV40 [27], efficient propagation of ww‐BKPyV has been achieved [21].

The aim of this study was to analyze the effects of the immunomodulatory cytokines IFN‐γ and TNF‐α on clinical ww‐ and rr‐BKPyV isolates. To this end, culture conditions of primary RPTEC allowing robust propagation of ww‐BKPyV were successfully established. Unexpectedly, this new assay revealed opposite effects of TNF‐α on ww‐ and rr‐strains. This result highlights the importance of studying clinically relevant ww‐BKPyV isolates for basic research and drug discovery, which is now possible, using the present replication assay.

## 2 Results

### 2.1 Replication of ww‐BKPyV in Primary RPTEC

**Figure 1:**
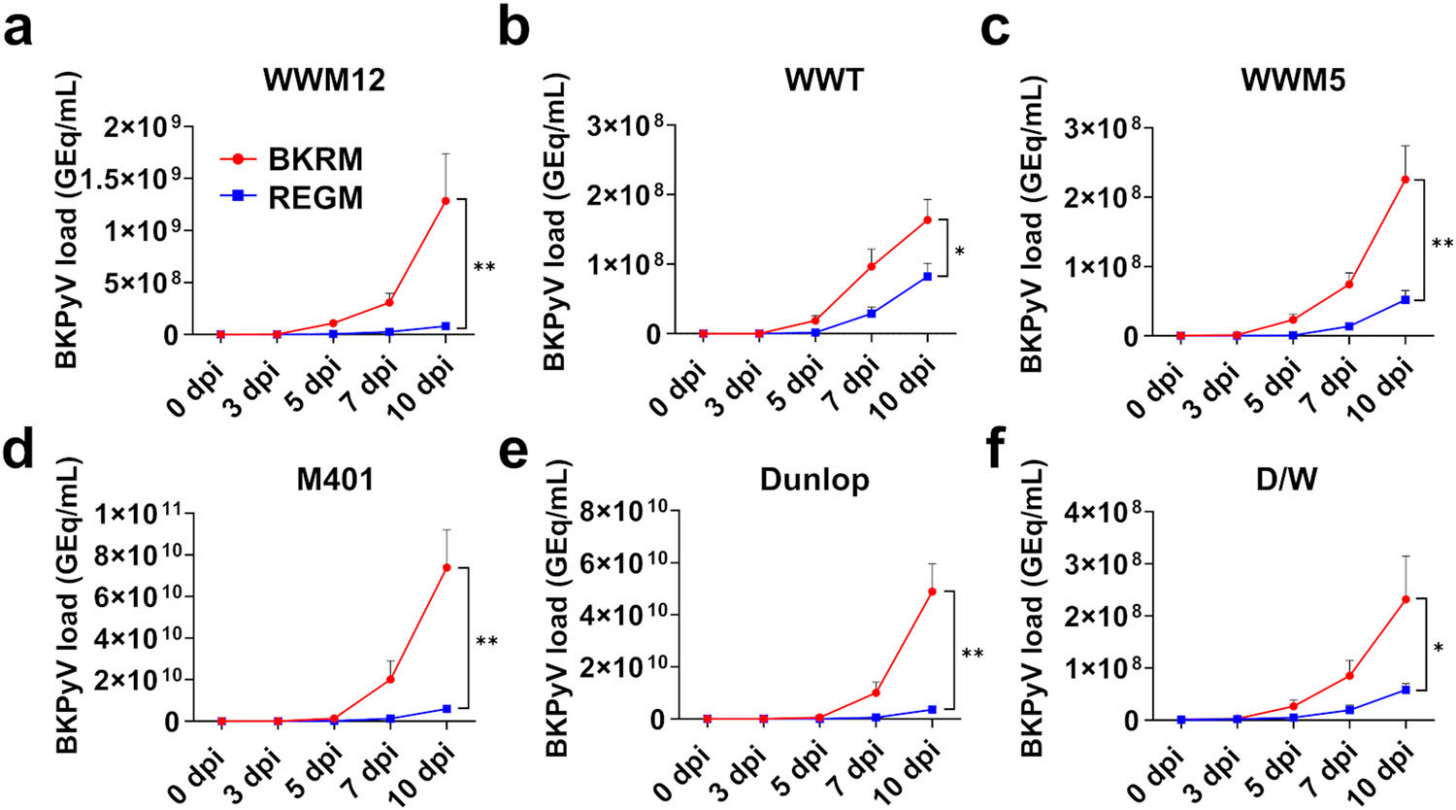
Effects of BKPyV Replication Medium (BKRM) on replication of ww‐ and rr‐BKPyV in RPTEC. RPTEC were seeded in BKRM or Renal Epithelial Growth Medium (REGM). One day later, they were infected with archetype (a: WWM12, b: WWT, c: WWM5) or partially rearranged (d: M401) BKPyV clinical isolates (multiplicity of infection [MOI] 0.02), with a clonal rr‐strain (e: Dunlop, MOI 0.02), or with the clonal recombinant D/W (f), in which the NCCR was exchanged for WWM12 NCCR in the Dunlop backbone (MOI 0.05). Viral loads in supernatants were measured at indicated days postinfection (dpi) by qPCR. The mean and standard error of the mean (SEM) of at least three independent experiments in technical triplicates is shown (Statistics: two‐way ANOVA). GEq/mL, genome equivalent per milliliter

To study clinical isolates of BKPyV, which are mostly archetypes, in primary kidney cells, culture conditions were optimized. FAD medium, classically used for keratinocyte culture [28], was found to support RPTEC cultivation for up to 12 passages. Flow cytometry or immunofluorescence staining for CD13, cubilin, megalin and gamma‐glutamyltransferase 1 (GGT1) confirmed the expression of typical kidney proximal tubule (PT) markers (Figure S1). The conditions for the replication of ww‐BKPyV were further improved, which resulted in a protocol using high‐passage RPTEC (typically passage 10–12), spinoculation (1 h, 1400 rpm, room temperature), and most importantly an optimized replication medium based on FAD, called BKPyV replication medium (BKRM, see Section 4). For BKPyV infection experiments, RPTEC were seeded in BKRM 1 day before infection and kept in this medium for the duration of the experiment (up to 10 days), which did not affect kidney PT marker expression (Figure S1). BKRM greatly enhanced the replication of three ww‐BKPyV clinical isolates (WWM12, WWT, and WWM5), compared with classical Renal Epithelial Cell Growth Medium (REGM) (Figure 1a–c). In addition, a clinical isolate called M401 was investigated. This strain contains 67.8% of rr‐strains as shown by next‐generation sequencing (NGS). The 10 most frequent clusters of the rearranged NCCRs (Figure S2b) have been aligned to the archetype and Dunlop strains (Figure S2a) and frequently contain insertions and deletions in the P, Q, and R blocks. In the RPTEC infection assay, BKRM also increased M401 replication (Figure 1d). Similar improvement was observed with two clonal strains: the laboratory‐adapted rearranged Dunlop strain and a recombinant BKPyV comprising the archetype WWM12 NCCR in the backbone of the Dunlop strain (D/W) (Figure 1e,f). Therefore, BKRM was used for all further experiments.

A common problem in previous attempts to cultivate ww‐BKPyV has been the emergence of NCCR rearrangements, which are responsible for an apparently improved replication compared with ww‐strains [24]. In the present assay, NGS analysis of viral NCCRs showed that, after 10 dpi in BRKM, the percentage of ww‐NCCR in the culture decreased by less than 1% compared with the respective inocula, for all ww‐strains (Table 1). Interestingly, when using the clonal strain D/W, virtually no NCCR rearrangements arose within 10 days of viral replication. The comparative analysis of point mutations, deletions and insertions between inocula and 10‐dpi samples did not reveal a unique pattern of selected rearrangements across 10‐dpi samples from different ww‐strains in BKRM (Figure S3). These data demonstrated that the novel BKPyV replication assay allows efficient replication of ww‐BKPyV in primary human RPTEC, with the vast majority remaining archetype during the tested 10‐day period.

**Table 1:** Percentages of ww‐NCCR in inocula and 10 dpi samples (BKRM) of ww‐strains shown in Figure 1.

	ww‐NCCR (%)	
Strain	Inoculum	10 dpi in BKRM^a^
WWM12	98.36	97.41 ± 0.89 (51)
WWT	97.77	97.43 ± 2.07 (3)
WWM5	99.18	98.44 ± 0.57 (2)
D/W	99.43	99.48 ± 0.05 (3)

a Mean ± SEM (number of independent experiments).

### 2.2 Characterization of ww‐ and rr‐BKPyV Replication in the Novel Replication Assay

**Figure 2:**
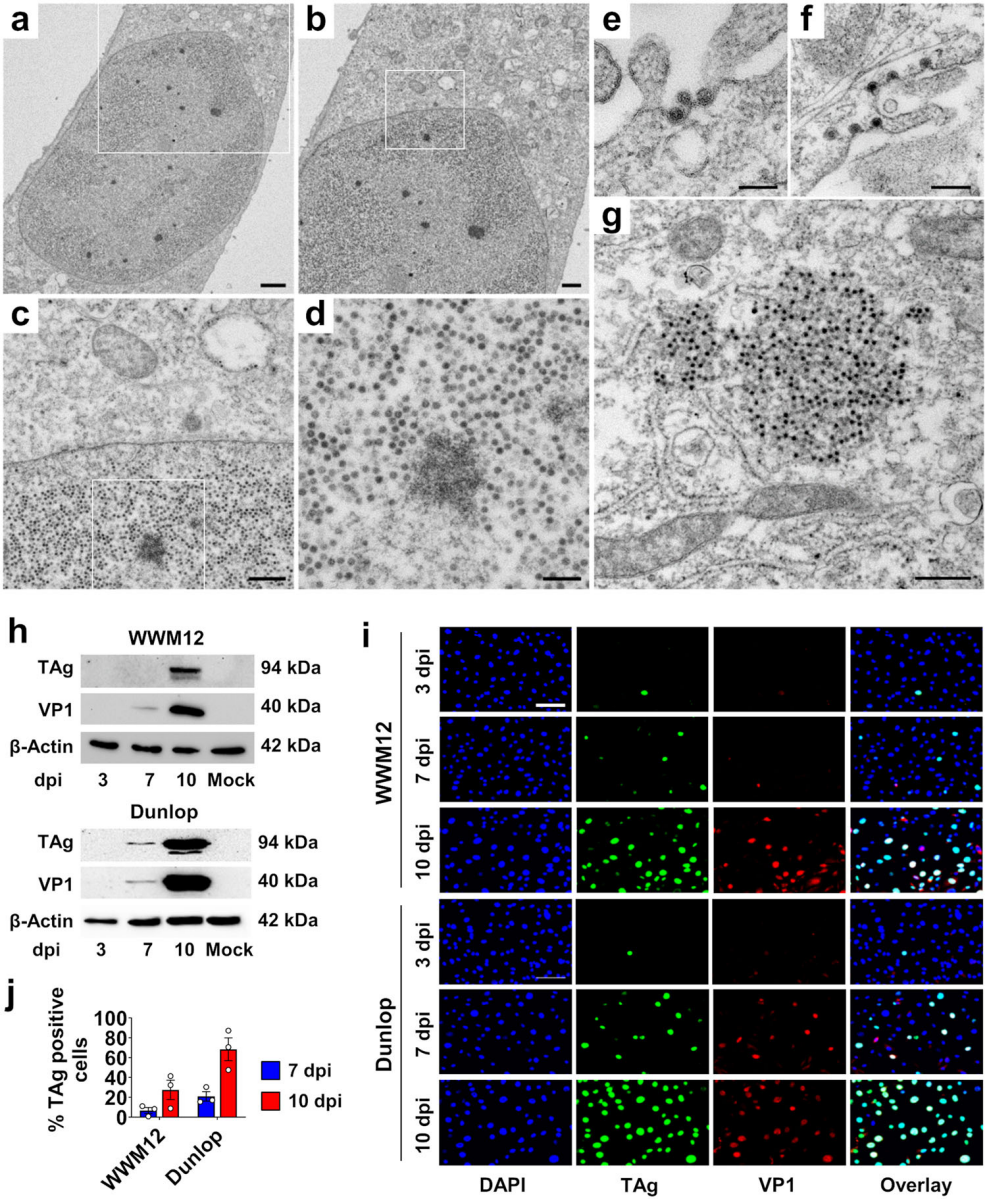
Archetypal BKPyV efficiently infects RPTEC. (a–g) WWM12‐infected RPTEC were fixed at 21 dpi and analyzed by TEM. The images (a–g) show viral particles in the nucleus of a single‐cell at increasing magnification (a–d), at the surface of cells (e), in endocytic compartments (f), and aggregated in the cytoplasm (g). Scale bars (a) = 2 µm, (b) = 1 µm, (c) = 500 nm, (d) = 200 nm, (e) = 100 nm, (f) = 200 nm, (g) = 500 nm. (h–j) TAg and VP1 expression was analyzed in WWM12‐ or Dunlop‐infected RPTEC at indicated dpi by immunoblot (loading control: β‐actin) (h) or by immunofluorescence (TAg: green, VP1: red, nuclei stained with DAPI, Scale bar = 100 µm) (i). One representative picture of three independent experiments is displayed in (i). (j) Quantification of TAg‐positive cells in immunofluorescence experiments (mean ± SEM of three independent experiments).

Transmission electron microscopy (TEM) of WWM12‐infected RPTEC under the new culture conditions demonstrated virion formation. Virus particles were present in large arrays within the nucleus of many cells (Figure 2a–d), which is the main compartment of BKPyV assembly [3]. They were also found at the surface of cells (Figure 2e), in endocytic compartments (Figure 2f), and aggregated in the cytoplasm (Figure 2g). The levels of the early and late viral proteins TAg and VP1 (respectively), detected by immunoblot in WWM12‐ or Dunlop‐infected RPTEC, increased over time and reached robust expression for both strains after 10 days. VP1 protein expression was first detected at Day 7 (Figure 2h and Figure S3) for both strains, while Dunlop TAg was detectable earlier (7 dpi vs. 10 dpi) and at higher levels than WWM12 TAg (Figure 2h and Figure S3). Similar TAg and VP1 expression kinetics were observed by immunofluorescence at the single‐cell level (Figure 2i). Quantification of TAg‐positive cells (Figure 2j) indicated that at 10 dpi, at least 68% and 27% of the cells were productively infected with Dunlop and WWM12, respectively. The differences observed at the protein level corresponded to an earlier increase in BKPyV DNA load in the supernatants of Dunlop compared with WWM12 infected RPTEC (3 vs. 7 dpi, Figure S3).

### 2.3 Opposing Effects of TNF‐α on ww‐ and rr‐BKPyV Strains

**Figure 3:**
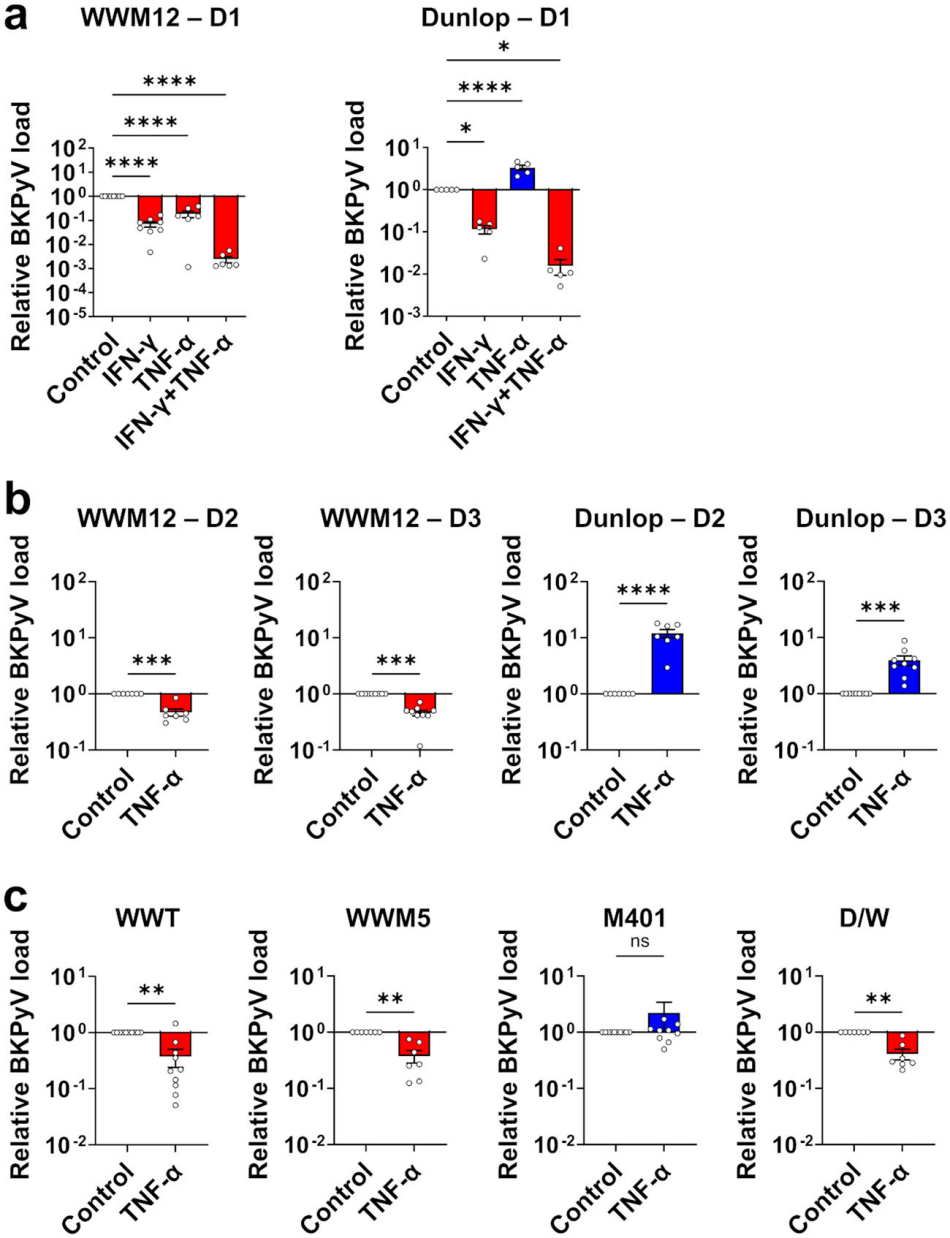
Effects of IFN‐γ and TNF‐α on replication of ww‐ and rr‐BKPyV strains. RPTEC were infected with the indicated strains (MOI 0.02, except D/W at MOI 0.05) and treated with 200 U/mL IFN‐γ, 1000 U/mL TNF‐α or both cytokines for 7 days. Viral replication was analyzed 7 dpi by qPCR on supernatants. RPTEC from different donors were used: donor 1 (D1) in (a) and (c); donors 2 and 3 (D2 and D3) in (b). The mean ± SEM of relative viral load normalized to the control at least 3 independent experiments in technical triplicates is shown. (Statistics: one‐way ANOVA with Dunnett's multiple comparisons test (a) or ratio paired *t* test (b, c)).

Taking advantage of the new ww‐BKPyV replication assay, the effects of the immunomodulatory cytokines IFN‐γ and TNF‐α on BKPyV replication were compared for the archetype WWM12 clinical isolate and the rearranged laboratory‐adapted Dunlop strain (MOI 0.02; Figure 3a). IFN‐γ significantly reduced the replication of both strains, confirming the results previously published for Dunlop [11] and extending them to ww‐BKPyV. TNF‐α alone promoted the replication of the rearranged Dunlop strain, congruent with previous reports [14]. IFN‐γ and TNF‐α together significantly reduced the replication of both strains, indicating that the presence of IFN‐γ dominated over or converted TNF‐α signaling. Surprisingly, TNF‐α alone significantly suppressed the replication of archetype WWM12 (Figure 3a).

The inhibitory effect of TNF‐α on the archetype replication was first observed at 5 dpi and further increased at Day 7. For the rearranged Dunlop strain, a weak positive effect of TNF‐α was observed already earlier at 3 dpi (Figure S4a,b).

The intriguing opposite responses of WWM12 and Dunlop to TNF‐α were then confirmed using two additional RPTEC donors (Figure 3b), higher MOIs (Figure S4a,b), also in additional BKPyV clinical isolates (Figure 3c) and with a broader range of TNF‐α concentrations (250–2000 U/mL), (Figure S4c). The ww‐isolates WWT and WWM5 were also significantly inhibited by TNF‐α, while the replication of the isolate M401 containing 67.8% of rr‐strains, was slightly increased (Figure 3c). This suggested that the type of NCCR (archetype or rearranged) may determine the viral response to TNF‐α stimulation. To examine the impact of the NCCR type on the response to TNF‐α, the recombinant chimeric D/W virus, in which the rr‐NCCR was replaced by the WWM12 ww‐NCCR in the context of the Dunlop backbone, was investigated. Interestingly, D/W behaved like other ww‐strains (Figure 3c), demonstrating that the effect of TNF‐α on viral replication depends on the type of NCCR.

**Figure 4:**
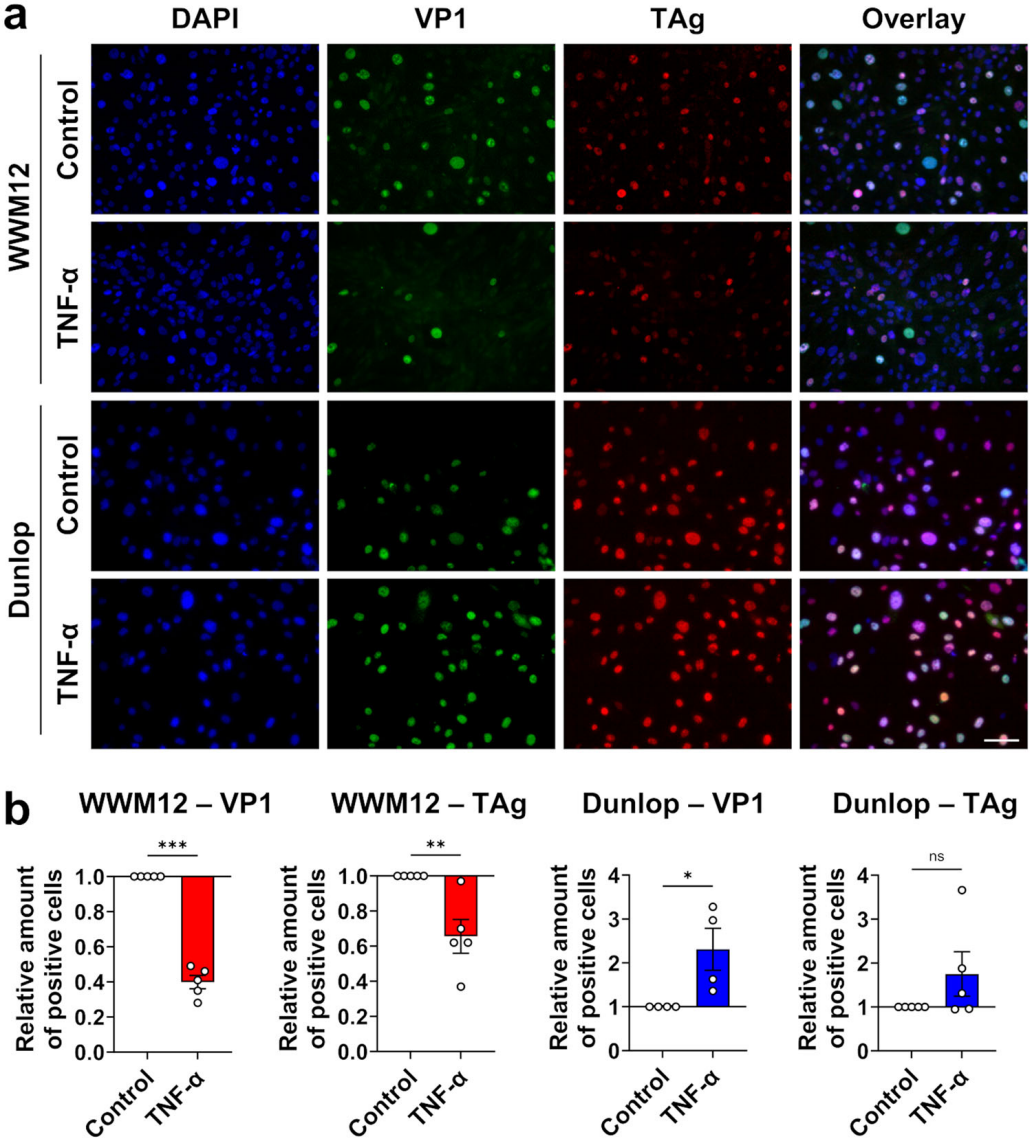
Influence of TNF‐α on ww‐ or rr‐BKPyV protein expression. (a) RPTEC infected with WWM12 or Dunlop (MOI 0.02) were treated with 1000 U/mL TNF‐α for 10 days. TAg (red) and VP1 (green) were then stained by immunofluorescence (nuclei stained with DAPI, scale bar = 100 µm). (b) Mean ± SEM of TAg and VP1 positive cells in at least three independent experiments. (Statistics: ratio paired *t* test).

Single‐cell immunofluorescence staining showed that TNF‐α significantly decreased VP1 expression in WWM12‐infected cells, whereas it significantly stimulated its expression in Dunlop‐infected cells (Figure 4), consistent with its effect on viral replication measured by qPCR (Figure 3a,b). The same trend was observed for TAg, but the effect was less pronounced and reached statistical significance only for WWM12.

Altogether, these data demonstrated that TNF‐α inhibits the replication of ww‐BKPyV, while it promotes rr‐BKPyV replication as shown in the new archetype replication assay with independent patient‐derived ww‐strains and RPTEC from independent donors.

### 2.4 Role of the IKK‐NF‐κB Pathway in the Effects of TNF‐α on BKPyV

**Figure 5:**
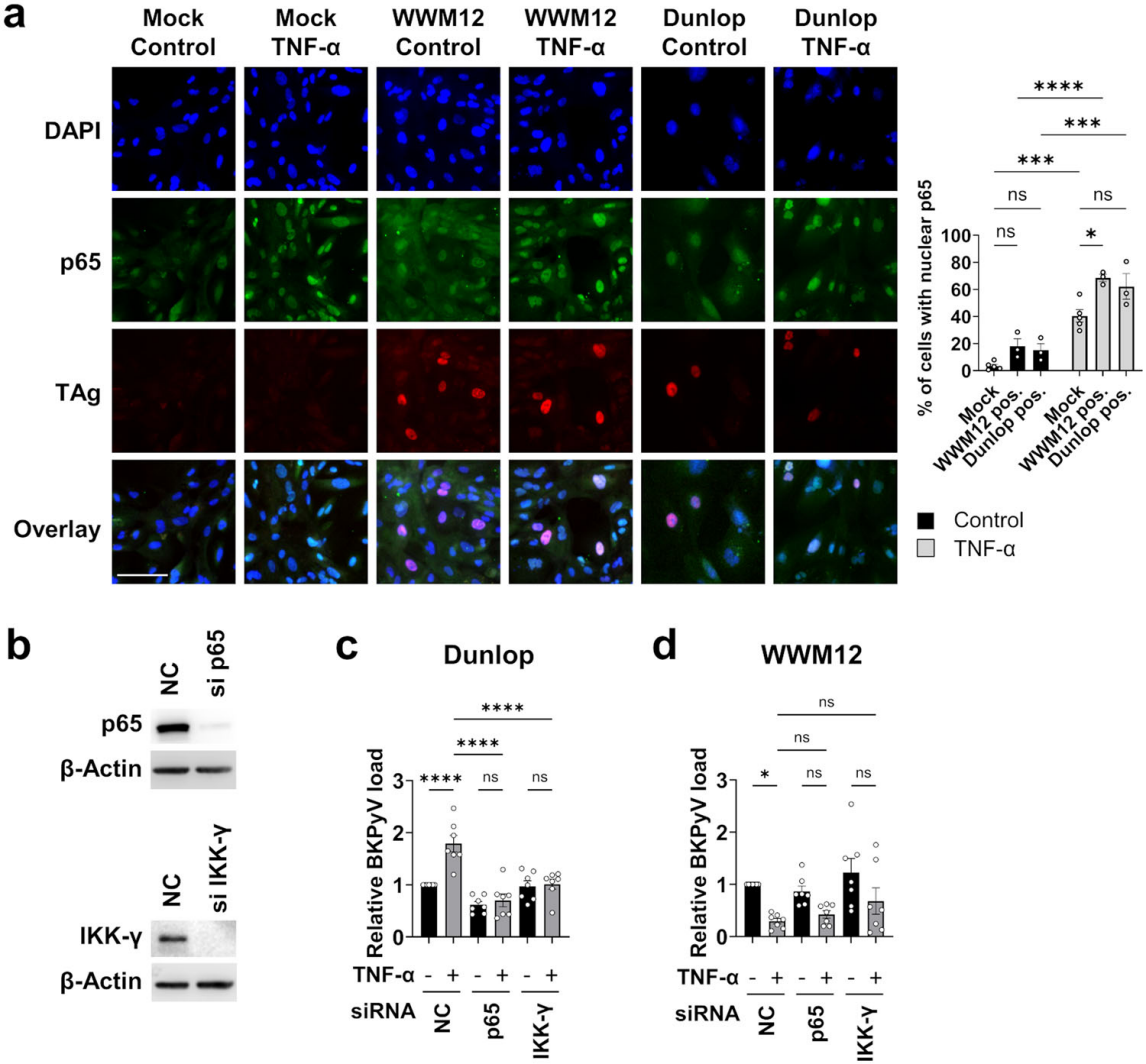
Role of the IKK‐NF‐κB pathway in the effects of TNF‐α on BKPyV. (a) RPTEC seeded in BKRM were infected or not with the indicated BKPyV strains (MOI 0.15) and stimulated 7 days later with 1000 U/mL of TNF‐α for 30 min or left untreated. BKPyV TAg expression and nuclear translocation of NF‐κB p65 were analyzed by immunofluorescence (green: p65, red: TAg, blue: DAPI, scale bar = 100 µm). The percentage of cells showing p65 nuclear localization was quantified in mock‐infected cells and in TAg positive WWM12 or Dunlop‐infected cells, in the unstimulated or TNF‐α‐stimulated conditions (right graph, mean ± SEM of at least three independent experiments, statistics: two‐way ANOVA with Šídák's multiple comparisons test). (b) Immunoblot analysis of p65 and IKK‐γ expression in RPTEC transfected with the indicated siRNAs for 11 days (NC: negative control siRNA). (c, d) RPTEC were transfected with the indicated siRNAs for 4 days before being infected with the indicated BKPyV strains (MOI 0.1). Viral replication was analyzed by qPCR 7 dpi. For each viral strain, the mean ± SEM of relative viral load normalized to the unstimulated NC siRNA condition of at least three independent experiments in technical triplicates is shown. (Statistics: one‐way ANOVA with Tukey's multiple comparisons test).

TNF‐α can stimulate various inflammatory signaling cascades including NF‐κB [12, 13], which was recently implicated in the induction of BKPyV replication by TNF‐α [14]. To investigate the role of NF‐κB for the present results, the ability of TNF‐α to induce the nuclear translocation of the p65 subunit of NF‐κB was first confirmed in RPTEC cultured in BKRM (Figure 5a, “Mock control” vs. “Mock TNF‐α”). In addition, p65 nuclear translocation was analyzed in ww‐ and rr‐BKPyV‐infected RPTEC, with or without TNF‐α stimulation. Interestingly, in the absence of TNF‐α stimulation, 18.1 ± 5.5% and 15.2 ± 4.8% of WWM12‐ and Dunlop‐infected cells (positive for TAg staining), respectively, were also positive for nuclear p65 (Figure 5a, WWM12 or Dunlop “control”), suggesting that BKPyV infection itself may activate the NF‐κB pathway. Upon TNF‐α stimulation, p65 was translocated to the nucleus in 40.4 ± 4.6% of uninfected cells and 68.5 ± 2.7% and 62.2 ± 9.5% of WWM12‐ and Dunlop‐infected cells (Figure 5a).

We then used several strategies to interfere with the NF‐κB pathway. First, we tested the widely used PS‐1145 inhibitor [29, 30, 31], which blocks the Inhibitor of NF‐κB (IκB) Kinase β (IKK‐β) [32]. PS‐1145 completely suppressed the TNF‐α‐induced increase of Dunlop replication (Figure S5a), as expected [14]. However, PS‐1145 also reverted the negative effect of TNF‐α on WWM12 replication in RPTEC in a dose‐dependent manner (Figure S5b). Parallel neutral red uptake assays showed that the effect of TNF‐α and PS‐1145 were not due to cytotoxic effects of the compounds (Figure S5c,d).

Since IKK‐β plays a role in the canonical but also noncanonical pathway and may even be involved in NF‐κB‐independent pathways [33], we then used siRNAs to silence the IKK subunit γ (IKK‐γ), an essential component of the canonical NF‐κB pathway, or the p65 subunit of NF‐κB, which showed strong nuclear translocation after TNF‐α stimulation. Immunoblot analysis confirmed efficient silencing of both proteins for up to 11 days posttransfection (Figure 5b), which is the duration of infection the experiment performed in Figure 5c,d. Similar to PS‐1145, siRNAs against p65 and IKK‐γ totally abrogated the increase of Dunlop replication by TNF‐α (Figure 5c), confirming the involvement of the IKK‐γ/NF‐κB p65 pathway in the activation of rr‐BKPyV replication. Parallel neutral red uptake assays showed no significant change in cell growth or viability in siRNA‐transfected cells (Figure S5e,f). The silencing of p65 and IKK‐γ only slightly and nonsignificantly reduced the inhibition of WWM12 by TNF‐α indicating that the IKK‐γ/NF‐κB p65 pathway may only partially contribute to TNF‐α‐mediated WWM12 repression. This suggested that a PS‐1145 activity unrelated to the canonical NF‐κB p65 pathway may play a more important role for ww‐BKPyV suppression.

NF‐κB could act directly via binding to the viral NCCR, or indirectly via the regulation of other cellular factors that modulate the replication of BKPyV. To investigate this question, an analysis of putative NF‐κB binding sites (BS) in the NCCR of ww‐ and rr‐strains was performed in silico using the online tool PROMO 3.0 [34, 35]. The NF‐κB BS in the O‐block, which we called BK1, had been described previously [16]. The BK1 core sequence is identical in the rearranged Dunlop strain and all investigated archetype strains (WWM5, WWM12, WWT). Moreover, 11 bp upstream and 68 bp downstream of this core sequence are also conserved. Compared with WWM12 there is only one T to G exchange 8 bp downstream of the core sequence present in WWM5, WWT, and Dunlop. Since WWM5 and WWT are suppressed by TNF‐α like WWM12, this mutation can, however, not account for the opposing response to Dunlop. A second site, pBK2, was predicted in the P‐block of all ww‐NCCRs as well as Dunlop. pBK2 is duplicated in Dunlop NCCR. An additional putative NF‐κB BS, pBK3D, was predicted in the Dunlop NCCR (Figure S2).

**Figure 6:**
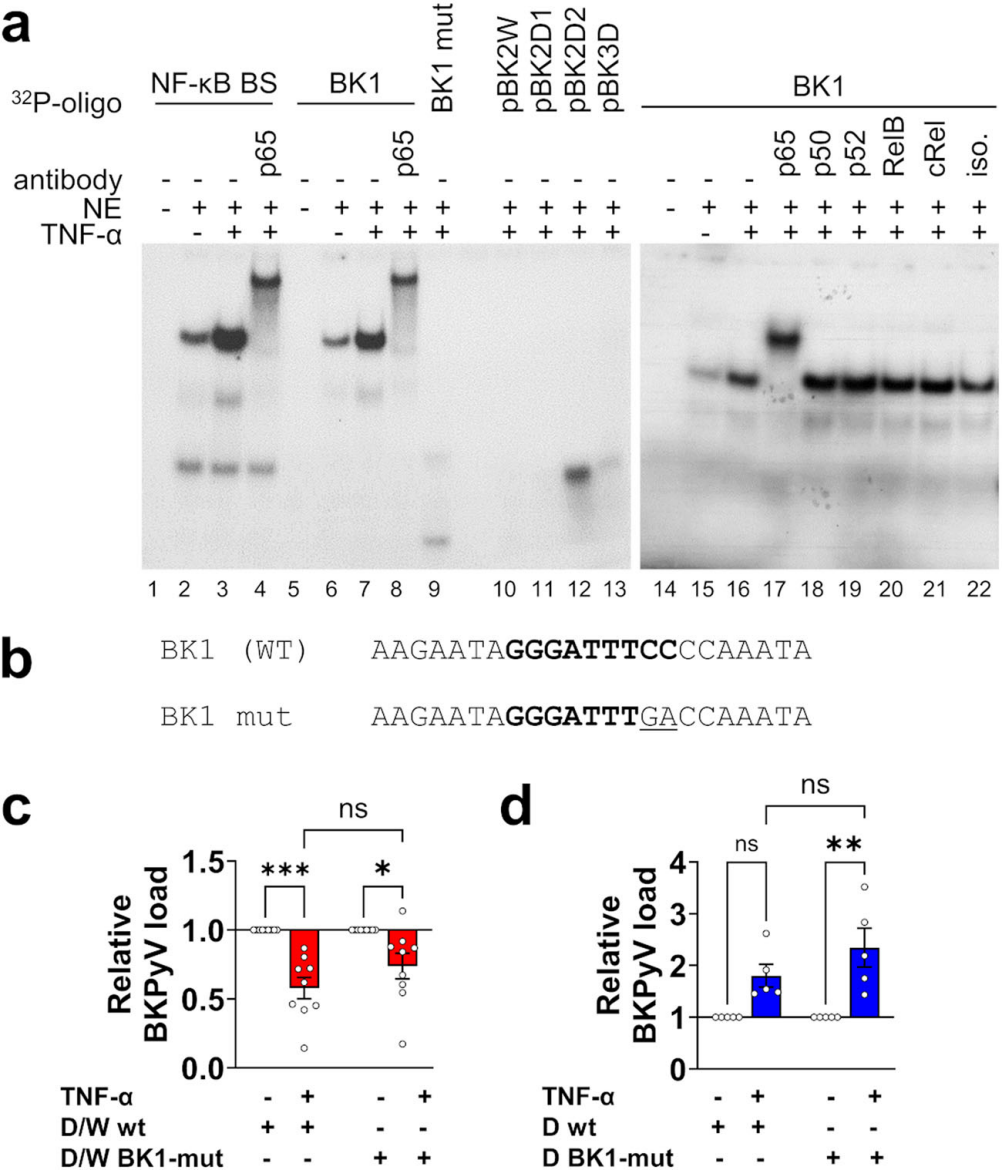
Impact of NF‐κB binding to BKPyV ww‐ and rr‐NCCRs on the opposing effects of TNF‐α. (a) Electrophoretic mobility shift assays (EMSA) were performed using ^32^P‐labeled double stranded DNA oligonucleotides containing NF‐κB BS from the mouse κ light chain enhancer (NF‐κB BS) or the NF‐κB BS predicted in BKPyV WWM12 or Dunlop NCCR (BK1, pBK2W, pBK2D1, pBK2D2, pBK3D, see Figure S2) incubated with nuclear extracts (NE) of RPTEC stimulated with 1000 U/mL of TNF‐α for 30 min, or unstimulated. When indicated, antibodies against NF‐κB subunits were added to the binding reaction (iso.: isotype control). (b) Sequence of the oligonucleotides containing the wild type (wt) BK1 binding site or the mutated BS. (c, d) RPTEC were infected with the clonal archetype D/W or rearranged Dunlop strains containing a wild type or mutated NF‐κB BS (MOI 0.1) and were stimulated with 1000 U/mL of TNF‐α or unstimulated. Viral replication was analyzed at 7 dpi by qPCR on supernatants. The mean ± SEM of relative viral load normalized to the control of at least three independent experiments in technical triplicates is shown (Statistics: two‐way ANOVA with Tukey's multiple comparisons test).

The ability of these (putative) BSs to bind NF‐κB was then tested in electrophoretic mobility shift assay (EMSA). Extracts from TNF‐α‐stimulated uninfected RPTEC showed increased binding activity over medium controls in the NF‐κB BS BK1 similar to an NF‐κB consensus BS [36]. The TNF‐α‐induced band was almost completely supershifted with an antibody against the NF‐κB subunit p65 but not p50, p52 or RelB NF‐κB subunits confirming that NF‐κB p65 binds to BS BK1 as previously described [16] (Figure 6a, left panel). Using the same extracts in the same EMSA as with BK1, no NF‐κB‐related band shifts were observed when oligonucleotides of the predicted NF‐κB BSs pBK2W (containing the pBK2 BS and surrounding 7 bp of the archetype NCCRs), pBK2D1 (containing the first pBK2 BS of Dunlop NCCR and surrounding 7 bp), pBK2D2 (containing the second and third pBK2 BS of Dunlop NCCR and surrounding 7 bp, which are identical for both BS), and pBK3D (containing the pBK3 BS of Dunlop NCCR and surrounding 7 bp) were used, indicating that these predicted putative BSs do not bind to NF‐κB (Figure 6a, right panel). The binding pattern did not change when extracts from TNF‐α‐stimulated cells previously infected with WWM12 or Dunlop were used (Figure S6).

We then investigated the involvement of the BK1 NF‐κB BS in the effect of TNF‐α on ww‐ or rr‐BKPyV. Mutation of 2 nt in BK1 (Figure 6b) within this NF‐κB BS completely abolished NF‐κB binding. Surprisingly, when these two mutations were introduced by site‐directed mutagenesis into the rearranged Dunlop (D BK1‐mut) or the chimeric recombinant Dunlop/WWM12 (D/W BK1‐mut) with ww‐NCCR, TNF‐α still significantly suppressed D/W BK1‐mut, although its effect was slightly diminished compared with the parental D/W strain (Figure 6c). Remarkably, also the Dunlop BK1‐mut was still activated by TNF‐α, even more so than the nonmutant strain (Figure 6d).

In summary, our data indicate that the canonical NF‐κB p65 pathway is central to the activating effect of TNF‐α on the rearranged strain Dunlop but not for its repressing activity on archetype strains. However, based on our results obtained with the Dunlop BK1‐mut, the activating effect of TNF‐α is not directly mediated via the conserved NF‐κB BS. This suggests that the rr‐NCCR has undergone an adaption to other NF‐κB‐dependent downstream pathways to overcome TNF‐α‐mediated suppression.

## 3 Discussion

In this study, a novel replication assay for ww‐BKPyV was developed, allowing to investigate, for the first time, the effects of immunomodulatory cytokines on clinical ww‐BKPyV isolates. With this assay, opposing effects of TNF‐α on ww‐ and rr‐BKPyV were clearly demonstrated. This study underscores the need to include clinically relevant ww‐BKPyV strains not only in basic BKPyV research and immunological studies but also for preclinical translational approaches to discover new anti‐BKPyviral drugs, since they may behave differently from rearranged strains.

This is the first report describing efficient in vitro replication of clinical archetype BKPyV isolates WWT, WWM5, WWM12 from different BKPyV genotypes Ib1, Ib2, or Ic in the natural host cells RPTEC. So far, only rearranged laboratory‐adapted forms like the Dunlop strain have been successfully propagated in primary RPTEC, whereas ww‐BKPyV replication was studied mainly in cell lines [21, 26]. Previous attempts to replicate ww‐BKPyV in primary RPTEC did not result in detectable viral replication, unless significant NCCR rearrangements occurred [22, 23, 24, 37]. Remarkably, NGS analysis proved that under the conditions of this novel assay, clonal archetype virtually retained the ww‐NCCR for at least 10 dpi, and in clinical archetype isolates, rearranged minority strains increased less than 1%, with no specific pattern of selected rearrangements at this stage (Table 1 and Figure S3).

Critical parameters facilitating archetypal BKPyV replication were identified: (1) usage of higher RPTEC passages, (2) centrifugation promoting cell‐virus interaction, and (3) a medium composition referred to as BKRM that provides a cellular environment favorable to archetypal replication. Higher cell passages may promote cellular senescence and higher expression of factors supporting polyomavirus replication, such as the kinases ATM and ATR [38], which is subject of ongoing studies. Importantly, RPTEC retained their renal phenotype under the conditions of this new assay (up to 12 passages in FAD medium and up to further 10 days after switching to BKRM) (Figure S1).

In immunocompetent patients, BKPyV is supposed to be controlled by cellular immunity. This includes T_H_1 cells, which respond to viral infection with a distinct cytokine response that typically comprises IFN‐γ and TNF‐α [9], and this is also the case for BKPyV infection [8]. The present data demonstrate that both cytokines, TNF‐α and IFN‐γ, together potently suppress BKPyV, irrespective of a ww‐ or rr‐NCCR, and this is also observed with IFN‐γ alone (Figure 3a), corresponding to the natural immune control of BKPyV infection in healthy individuals.

When cellular immunity is suppressed after renal transplantation [39, 40], this T_H_1‐derived IFN‐γ‐comprising antiviral response is severely limited, while the pro‐inflammatory cytokine TNF‐α can still be produced in high amounts by injured or infected epithelial cells and macrophages in the local microenvironment. However, the impact of TNF‐α on the naturally transmitted ww‐BKPyV, had not been studied. The new ww‐BKPyV replication assay allowed to show that TNF‐α inhibits replication of all clinical ww‐isolates tested (Figures 3 and 4). This inhibitory effect was first observed at 5 dpi and further increased at Day 7 suggesting that continuous cycles of viral replication amplify the differences over time.

This was in strong contrast to the replication of rr‐BKPyV, which was increased by TNF‐α already at 3 dpi in line with published results [14]. Although the strain used by Li et al. [14] was not clearly specified, in view of the present data, their assay setup and of the former difficulties to replicate ww‐strains in vitro, it can be hypothesized that the authors have used a rr‐strain.

In kidney transplant patients, elevated TNF‐α urine levels are observed in PVAN, and this often precedes the onset of PVAN [14, 41]. Moreover, rearranged strains are more often detected in PVAN patients. The present data lead to the hypothesis that under immunosuppressive conditions, TNF‐α might exert pressure on the transmitted archetype. Archetype is inhibited, thereby favoring in‐host evolution of rr‐BKPyV or providing a selective advantage for rr‐BKPyV minority strains, possibly explaining the emergence and selection of rearranged, TNF‐α‐resistant or ‐promoted strains during the disease course in patients. These rearranged strains may have an increased replication capacity and cytopathogenicity [17], thereby causing severe kidney damage leading to PVAN. Further studies are required to confirm this hypothesis. In‐host evolution of BKPyV through APOBEC3 driven mutagenesis has previously been demonstrated in renal transplant recipients, and in the setting of full‐blown PVAN [42]. Intriguingly, APOBEC3 is typically induced in the context of inflammation, which may add further complexity to a potential inflammation‐associated BKPyV in‐host evolution.

TNF‐α also inhibited the replication of the clonal recombinant D/W strain, containing the archetype WWM12 NCCR in the context of Dunlop genome. This clearly shows that the NCCR, which comprises the viral promoters and enhancers, is the main determinant of the viral response to TNF‐α. Such a differential responsiveness of ww‐ or rr‐BKPyV promoters is not unprecedented, since it had previously been observed in response to another cytokine, transforming growth factor‐β [43].

Upon receptor binding, TNF‐α can activate several pathways including the NF‐κB pathway, leading to cellular gene regulation [12, 13]. Notably, the small molecule inhibitor PS‐1145 targeting IKK‐β not only abolished the positive effect of TNF‐α on rr‐BKPyV replication but it was also able to fully rescue the replication of ww‐BKPyV in the presence of TNF‐α. In contrast, a differential effect on rr‐ and ww‐BKPyV was observed with siRNAs targeting IKK‐γ, which is an essential component of the canonical NF‐κB pathway, or the downstream NF‐κB subunit p65. Both siRNAs abolished the positive effect of TNF‐α on rr‐BKPyV replication but had only a minor effect on the repression of ww‐BKPyV. These results demonstrated that the IKK‐γ‐dependent canonical NF‐κB p65 pathway is central for the TNF‐α‐induced enhancement of rr‐BKPyV replication in line with previous results from Li et al. [14], but has only a minor role in TNF‐α‐mediated ww‐BKPyV suppression. The strong effect of PS‐1145 on ww‐BKPyV might be explained by the fact that IKK‐β is not only involved in the canonical and noncanonical NF‐κB pathways but also in various other signaling pathways, such as Foxo3a, Bcl‐10 and also MAP kinase pathways [33], that could play a role in ww‐BKPyV suppression. However, further research is needed to answer this question, as well as to determine whether these pathways directly suppress viral transcription from the ww‐NCCR or act indirectly via the regulation of other cellular factor(s).

In silico and experimental electromobility shift analyses revealed the presence of one conserved NF‐κB BS, which was previously described [16], in the NCCRs of all ww‐ and rr‐BKPyV strains. TNF‐α increased the binding activity of the NF‐κB p65 subunit at the conserved site but not of other subunits. Other predicted BS did not show NF‐κB binding activity, and this was the same in ww‐ or rr‐BKPyV infected cells.

Mutation of 2 nt within this NF‐κB BS completely abolished NF‐κB binding. Surprisingly, introduction of these mutations only slightly attenuated TNF‐α‐mediated suppression of the ww‐BKPyV, and did not affect the TNF‐α‐mediated activation of rr‐BKPyV replication indicating that the NF‐κB BS was not critical for the opposing effects of TNF‐α.

Our results indicate that TNF‐α inhibits the replication of ww‐BKPyV via an IKK‐β‐dependent mechanism, which could affect any stage of the viral life cycle. In patients with impaired T_H_1 immunity, this pressure may lead to the selection of BKPyV strains, which are rather promoted by TNF‐α. Since the NCCR is the determinant for the switch from the negative to positive impact of TNF‐α on the virus, we compared the NCCRs of the well‐described Dunlop and the predominant rearranged strain of the patient‐derived M401 (M401‐ID00004, Figure S2), which are both promoted by TNF‐α, with the ww‐BKPyV strains. One of the most obvious differences in Dunlop are AP‐1 BS that were newly created at the junctions of adjacent sequence repeats [44]. In silico analysis also predicted increased numbers of putative AP‐1 BS in the NCCR of predominant rearranged strain of the patient‐derived M401 (Figure S2). Notably, AP‐1 not only activates the BKPyV early promoter [44] but its expression and activity is also regulated in an NF‐κB‐dependent manner [45]. However, this does not exclude that NCCR alterations in BS for other BKPyV regulating transcription factors, that are directly or indirectly activated via the NF‐κB p65 pathway, may play a role. Further studies will be needed to investigate these complex mechanisms.

Based on their published data, Li et al. [14] suggested to explore the therapeutic effects of TNF‐α blockade in clinical trials. The data shown in this study strongly suggest a more cautious view, as TNF‐α pathway inhibitors may have opposite effects on ww‐BKPyV strains. Archetype BKPyV are the most common strains and still dominate in 50% of PVAN patients [17]. Even in most patients with rr‐BKPyV, a proportion of ww‐BKPyV is present [18]. In all these cases, the application of TNF‐α pathway inhibitors may lead to ww‐BKPyV escape from the natural antiviral immune control of TNF‐α.

In summary, these results reveal for the first time fundamental differences of ww‐ versus rr‐BKPyV replication. They demonstrate that TNF‐α inhibits ww‐BKPyV replication, whereas it promotes rr‐BKPyV replication. Our data indicate that NCCR rearrangements, which create a positive feedback to pathways downstream of NF‐κB p65 signaling, may overcome the initial TNF‐α‐mediated suppression of ww‐BKPyV. This highlights the importance of validating former and future results that were obtained with rearranged BKPyV laboratory strains on ww‐BKPyV clinical isolates, to draw clinically relevant conclusions, especially regarding antivirals. In this view, this novel assay paves the way for in‐depth analysis of the ww‐BKPyV life cycle and of novel antivirals against relevant ww‐BKPyV strains in their natural host cells.

## 4 Methods

### 4.1 Cells and Media

Healthy cortical kidney tissue (Urology Department, Saarland University Medical Center), was cut and digested in RPMI‐1640 containing 10% bovine serum albumin (BSA) and 1% collagenase for 1 h at 37°C. Digested cells were washed with Dulbecco's phosphate buffered saline (DPBS; without calcium and magnesium), resuspended in Trypsin/EDTA solution, and incubated for 30 min at 37°C. The digestion was stopped with DPBS containing 10% fetal calf serum (FCS). The suspension was filtered through 100, 70, and 40 µm cell strainers (BD Falcon) and plated in RPMI‐1640 containing 10% FCS.

For immunomagnetic separation of RPTEC, the cells were detached by Trypsin/EDTA and washed with wash buffer (DPBS, 0.5% BSA, 2 mM EDTA). The pellet was resuspended in 100 µL wash buffer with 5 µg mouse anti‐CD13 per 10^7^ cells and incubated for 30 min on ice. After three washes, the cells were resuspended in 80 µL wash buffer and 20 µL mouse anti‐IgG1 MACS‐microbeads (Miltenyi Biotech). After 15 min incubation on ice followed by three washes, labeled RPTEC were separated using MACS MS or LS columns and a MACS separator (Miltenyi Biotech). RPTEC were grown and passaged in FAD‐medium [28] containing Dulbecco's Modified Eagle's Medium (DMEM)/Ham's Nutrient Mixture F12 (3:1) supplemented with 10% FCS, penicillin (100 U/mL), streptomycin (0.1 mg/mL), hydrocortisone (0.4 µg/mL), cholera toxin (0.1 nM), epidermal growth factor (10 ng/mL), insulin (5 µg/mL), triiodothyronine (0.02 nM), adenine (0.18 mM), and transferrin (5 µg/mL) (all from Sigma‐Aldrich).

### 4.2 Preparation of BKPyV Stocks

To propagate the patient‐derived BKPyV strains WWM12, WWT, WWM5 and M401, HEK 293TT cells seeded in DMEM containing 10% FCS, 1% penicillin/streptomycin, 1% sodium pyruvate, and 400 µg/mL hygromycin B were infected with urine samples from patients of the Saarland University Medical Center, Homburg, Germany, with high BKPyV loads (> 10^7^ GEq/mL). After 1‐h incubation at 37°C, the viral inoculum was removed; the cells were washed with DPBS and cultivated in fresh medium. Supernatants were collected weekly, centrifuged and the cell‐free supernatant was stored at −80°C.

To produce the clonal Dunlop or D/W BKPyV stocks or the respective mutants (mut) for the NF‐κB BS BK1, BKPyV genome was excised by BamHI digestion from pBR322 BK Dunlop wt or mut or from pBR322 D/W wt or mut plasmids, respectively, religated using T4 ligase and transfected into HEK 293TT cells using Lipofectamine 2000 (Thermo Fisher Scientific). Supernatants were handled as described above.

Viral loads were measured by quantitative real‐time PCR (qPCR, see Supplementary Methods). The type of NCCR, and percentage of ww‐NCCR were validated by NGS (see Supplementary Methods). All ww‐stocks contained more than 97% ww‐NCCR. The M401 stock contained a mixture of 32.2% ww‐ and 67.8% rr‐BKPyV strains.

The genotypes of the patient‐derived strains were determined as Ib1 for WWM12 and M401, Ib2 for WWT, and Ic for WWM5 (see Supplementary Material for additional information on genotype determination).

### 4.3 Focus Forming Assay (FFA)

Infectious titers of BKPyV stocks were determined by a FFA adapted from [46]. RPTEC seeded on coverslips were infected with BKPyV. TAg was stained 5 dpi with anti‐SV40 TAg and a FITC‐labeled secondary antibody. At least 10 random pictures per condition were taken and infectious titers were calculated based on TAg positive cells. The mean of three experiments was calculated.

### 4.4 BKPyV Infections

RPTEC of passages 10–12 were seeded in 96‐well plates (2 × 10^5^ cells/well) in 200 µL of BKRM containing DMEM/Ham's Nutrient Mixture F12 (3:1) supplemented with 10% FCS, penicillin (100 U/mL), streptomycin (0.1 mg/mL), cholera toxin (0.1 nM), insulin (5 µg/mL), and triiodothyronine (0.02 nM) 1 day before infection and cultured therein after the infection. In Figure 1, BKRM was compared with REGM (Lonza Bioscience) as indicated in the legend. Unless otherwise stated, infections were performed in DMEM without supplements at a MOI of 0.02 infectious units/cell, as determined by FFA. The cells were centrifuged with the viral inoculum for 1 h at 280*g*. After three washes in PBS, fresh BKRM (or, when indicated in Figure 1, REGM) was added. When indicated, cytokines or compounds were added to the BKRM. Infected cells were cultivated for the indicated time intervals and the viral DNA in the supernatant was isolated with the NucliSENS easyMAG system (bioMérieux) or with the MagNA Pure 96 System (Roche) and quantified by qPCR (see Supplementary Methods).

### 4.5 Cytokines and Compounds

TNF‐α (Boehringer Ingelheim) was solved in DMEM with 10% sodium pyruvate and used at a final concentration of 1000 U/mL. IFN‐γ (Pepro Tech) was solved in PBS and used at a final concentration of 200 U/mL. PS‐1145 (Selleckchem) was solved in DMSO. Within each experiment, matched final DMSO concentrations were used (typically 0.1%).

Plasmids, Viral load determination by quantitative PCR, PCR amplification and NGS analysis of BKPyV NCCR, Flow cytometry, Immunoblot, Immunofluorescence, Transmission Electron Microscopy (TEM), siRNA transfection and Electrophoretic mobility shift assay (EMSA): See Supplementary Methods.

### 4.6 Statistical Analysis

Statistical tests were performed on at least three independent experiments, using GraphPad Prism 9.5.0. Means of technical replicates of each independent experiment were used for statistical tests as indicated in the respective figure legends. Statistical significances: ****p < 0.0001, ***p < 0.001, **p < 0.01, *p < 0.05, ns: not significant.

## Author Contributions

Sigrun Smola and Lise Lauterbach‐Rivière designed the study. Martin Janssen, Michael Stöckle, David Schmit, and Danilo Fliser provided primary specimens. Lise Lauterbach‐Rivière, Lucia Thuringer, Pascal Feld, Lina Kathrin Toews, Jessica Schüssler, Lars Gläser, Jonas Klinz, and Anna Sternjakob performed experiments. Lars Möller and Michael Laue performed TEM analysis. Marcel A. Lauterbach, Gilles Gasparoni, Kathrin Kattler‐Lackes, and Sven Rahmann performed bioinformatics analysis, Lise Lauterbach‐Rivière, Lucia Thuringer, Stefan Lohse, and Sigrun Smola analyzed the data. Lise Lauterbach‐Rivière, Pascal Feld, and Sigrun Smola prepared the manuscript with input from the other authors.

## Ethics Statement

Approval for the use of human kidney tissue and human urine samples was obtained from the Ethics committee of the Medical Association of Saarland (votes 14/13 and 36/22). All methods were carried out in accordance with relevant guidelines and regulations.

## Conflicts of Interest

The authors declare no conflicts of interest.

## Materials and Correspondence

All unique biological materials used in this study are available from the corresponding author upon request. Correspondence and material requests should be addressed to Prof. Sigrun Smola: sigrun.smola@uks.eu.

## Supporting information

Supporting information.

## Data Availability

All data that support the findings of this study are available from the corresponding author upon reasonable request.
