# Supplementary material for: Tumor Necrosis Factor‐Alpha Inhibits the Replication of Patient‐Derived Archetype BK Polyomavirus While Activating Rearranged Strains
Source: J Med Virol. 2025 Feb 14;97(2):e70210. doi: 10.1002/jmv.70210 (PMC11826303; doi:10.1002/jmv.70210)
Supplement: Supplementary file 1 — Supporting information. [file JMV-97-e70210-s001.docx]

Supplementary Materials for

Tumor Necrosis Factor-alpha inhibits the replication of patient-derived archetype BK polyomavirus while activating rearranged strains

**Authors**

Lise Lauterbach-Rivière^1#^, Lucia Thuringer^1,2#^, Pascal Feld^1#^, Lina Kathrin Toews^1^, Jessica Schüssler^1^, Jonas Klinz^1^, Lars Gläser^1^, Stefan Lohse^1^, Anna Sternjakob^1^, Gilles Gasparoni^3^, Kathrin Kattler-Lackes^3^, Jörn Walter^3^, Marcel A. Lauterbach^4^, Sven Rahmann^5^, Lars Möller^6^, Michael Laue^6^, Martin Janssen^7,§^ , Michael Stöckle^7^, David Schmit^8^, Danilo Fliser^8^, Sigrun Smola^1,2^*

**Affiliations**

^1^Institute of Virology, Saarland University Medical Center, D-66421 Homburg, Germany

^2^Helmholtz Institute for Pharmaceutical Research Saarland (HIPS), Helmholtz Centre for Infection Research, Saarland University Campus, D-66123 Saarbrücken, Germany

^3^Department of Genetics, Saarland University, D-66123 Saarbrücken, Germany

^4^Molecular Imaging, Center for Integrative Physiology and Molecular Medicine, Saarland University, D-66421 Homburg, Germany

^5^Algorithmic Bioinformatics, Center for Bioinformatics Saar, Saarland Informatics Campus, Saarland University, D-66123 Saarbrücken, Germany

^6^Advanced Light and Electron Microscopy, Centre for Biological Threats and Special Pathogens, Robert Koch-Institute, D-13353 Berlin, Germany

^7^Department of Urology, Saarland University Medical Center, D-66421 Homburg, Germany

^8^Department of Nephrology, Saarland University Medical Center, D-66421 Homburg, Germany

# equal contribution

*To whom correspondence should be addressed: [sigrun.smola@uks.eu](mailto:sigrun.smola@uks.eu)

^§^Present address: Department of Urology, University Hospital Muenster, Albert-Schweitzer Campus 1 A1, 48149, Muenster, Germany

**Supplementary Methods**

*Plasmids*

The pBR322 Dunlop plasmid was a gift from Peter Howley (Addgene #25466) and was used to produce the Dunlop BKPyV stocks. The pBR322 Dunlop/WWM12 plasmid, used to produce the recombinant D/W BKPyV stocks, was produced by exchanging the Dunlop NCCR for WWM12 NCCR by overlap-extension PCR [1]. Briefly, WWM12 NCCR was amplified from DNA isolated from WWM12 viral stocks using the primers P3 5’–GCTGGCGCAGAACCATGGCCTTTGTCCAGTTTAAC–3’ and P4 5’– GTTAAGAACTTTATCCATTTTTGCAAAAATTGC–3’. The PCR product was used as megaprimers in a second PCR to amplify the pBR322 BK Dunlop plasmid backbone. The PCR product was digested with DpnI to remove the original plasmid, and subsequently transformed into competent bacteria. The integrity of the resulting pBR322 Dunlop/WWM12 plasmid isolated from a clone was verified by Sanger sequencing of the full-length plasmid. A mutation in the NF-κB BS BK1 (Fig. 6b) was introduced into pBR322 Dunlop and pBR322 Dunlop/WWM12 using the Q5 site directed mutagenesis kit (New England Biolabs) and the following primers, respectively: Dmut_F: 5’-AACTATTTGGTCAAATCCCTATTCTTTTGC-3’, Dmut_R: 5’-TTGCTAGGCCTCAGAAAAAG-3’ (for pBR322 Dunlop) and Wmut_F: 5’-CTAGGCCTCAGAAAAAGCCTCC-3’, Wmut_R: 5’-CAAAAATATTTGGTCAAATCCCTATTC-3’ (pBR322 Dun/WWM12).

*Viral load determination by quantitative PCR (qPCR)*
 For viral load measurements, qPCR was performed with a plasmid standard curve using the primers VP2-F (5‘-CCCCAGGAGGTGCTAATC-3‘) and VP2-R (5‘-CAGTCCCGTACAGGCCTAGAA-3‘), a hydrolysis probe (5‘-6FAM-AAGAACTGCTCCTCAATGGATGTTGCCTT--TMR-3‘) and the Light Cycler Fast Start DNA MasterPLUS Hybprobe kit (Roche, Mannheim, Germany) according to the manufacturer’s instruction, on a Light Cycler 480 II (Roche, Mannheim, Germany) using the following program: 95°C for 15 min, 50 cycles with 95 °C for 15 sec and 61 °C for 25 sec.

*BKPyV genotype determination*

To determine the genotype of the patient-derived BKPyV strains, a nested PCR was performed on viral DNA extracted with the MagNA Pure 96 System (Roche), using the primers BKV-S1920 5’-ATC AAA GAA CTG CTC CTC AAT-3’ and BKV-A2159 5’-GCA CTC CCT GCA TTT CCA AGG G-3’ for the outer PCR and BK_ 327-1 5’-CAA GTG CCA AAA CTACTA AT-3’ and BK_ 327-2 5’-TGC ATG AAG GTT AAG CAT GC-3’ for the inner PCR (Boldorini et al 2009) and the following PCR program: denaturation 30 sec at 98°C; 35 cycles (outer PCR) or 30 cycles (inner PCR) with denaturation 10 sec at 98°C, annealing 20 sec at 55°C, elongation 20 sec at 72°C; final extension of 2 min at 72°C. The Q5 Hot Start High-Fidelity DNA Polymerase (New England Biolabs) was used. In order to further distinguish the subgenotypes within genotype I, a second VP1 region was amplified by nested PCR using the primers BK_228 5’-GAATAACTAGCATGCTTAACC-3’ and BK_229 5’-CACAAATATCAGCAGCTG-3’ for the outer PCR and BK_225 5’-GTAATTTCCACTTCTTTGCTG-3’ and BK_227 5’-GGTCACATGAAGTACTGGGGG-3’ for the inner PCR and the following PCR program: denaturation 30 sec at 98°C; 35 cycles (outer PCR) or 30 cycles (inner PCR) with denaturation 10 sec at 98°C, annealing 20 sec at 59°C (outer PCR) or 58°C (inner PCR), elongation 20 sec at 72°C; final extension of 2 min at 72°C. The PCR products were cleaned up using NucleoSpin™ Gel and PCR Clean-up Kit (Macherey-Nagel, Düren, Germany) and Sanger sequenced by Eurofins Genomics (Ebersberg, Germany) using the primers used for the inner PCRs. To determine the genotype, the sequences were compared with the following reference sequences (GenBank accession numbers): genotype Ia1: DQ989807.1 ; Ib1: AB211374.1 ; Ib2: DQ989795.1 ; Ic: AB369099.1; II : AB263920.1; III : AB365130.1 ; IVa1 : AB269869.1 ; IVa2 : AB211389.1 ; IVb1 : AB211390.1 ; IVb2 : AB211388.1 ; IVc1 : AB269827.1 ; IVc2 : AB260033.1.

*NGS analysis of BKPyV NCCR*

For analysis of BKPyV NCCR, viral DNA was isolated using the NucliSENS easyMAG system (bioMérieux, Nürtingen, Germany) and the viral NCCR was amplified with primers adapted from Gosert et al. [2] containing unique molecule identifiers (UMI) and NGS adapters (BKTT7_UMI_NGS_F and BKTT8_UMI_NGS_R, Table S1), using Q5 Hot Start High-Fidelity DNA Polymerase (New England Biolabs) and the following PCR program: denaturation 1 min at 98°C; 45 cycles with denaturation 10 sec at 98°C, annealing 90 sec at 61°C, elongation 25 sec at 72°C; final extension of 2 min at 72°C. The amplicons were purified using SPRIselect beads (Beckman Coulter) with a ratio beads/sample of 0.9 to remove dimers of primers. The DNA concentration was then determined using a Qubit 4 fluorometer and the Qubit™ dsDNA HS Assay Kit (Invitrogen).

To avoid any bias due to preferential sequencing of short reads, a length standard was generated by PCR amplification of archetype NCCR DNA fragments of known length using pBR322 Dunlop/WWM12 plasmid as template, the eleven forward primers listed in Table S1 and the BKTT8_UMI_NGS_R reverse primer (Table S1) with the same PCR conditions as above. The amplicons were run on a 2% agarose gel and extracted using the MinElute gel extraction kit (Qiagen). After determination of the DNA concentration using a Qubit 4 fluorometer, the eleven fragments were mixed in a known ratio. The standard fragments were prepared in triplicate.

All amplified products (samples and standards) were diluted to 4 nM and NGS tags were finalized by a second PCR step (5 cycles) with NGS adaptor tagging primers, followed by a final clean-up (Agencourt AMPure XP Beads). Amplicons were diluted, pooled and sequenced (v3 chemistry: 2 × 300 bp paired-end) on the Illumina MiSeq following the manufacturer’s instructions aiming at 100,000 reads per amplicon.

The raw data was quality checked using FastQC and trimmed for adaptors or low quality bases using the tools cutadapt and Trim Galore!. Paired-end reads were merged with the FLASh tool. Further bioinformatics analysis was performed with the Matlab software (Mathworks, Natick, USA). Samples with an overlap larger than 10 nucleotides between 5’ and 3’ reads and a total length of more than 250 nucleotides were selected. Unique molecules were identified based on UMIs. To identify mutations, insertions and deletions as well as to determine the archetype or rearranged structure of the NCCR, the reads were aligned to the respective archetype NCCR sequence (GenBank reference sequences JQ513603.1 (WWM12), M34048.1 (WWT) or JQ513596.1 (WWM5)) using the Needleman-Wunsch algorithm (Matlab function nwalign with gapopen penalty 10, extendgap penalty 1). Reads with deletions, insertions or mutations longer than 2 nucleotides were classified as rearranged. The length standards were used to fit a function describing the ratio between the actual number of reads and the theoretical number of reads (if sequencing efficiency was not depending on sequence length) as a function of sequence length. The frequency of each sequence was corrected according to its length using this length standard function and the corrected percentage of archetype sequences was then determined.

To identify the representative NCCR sequences of the rr-strains of M401, pairwise jump distances were computed between all rr-sequences. The average of the jump distances between two sequences was used for performing single-linkage clustering of the sequences using a threshold of T=2. The representative sequences of the 10 most frequent clusters have been aligned to the NCCR sequences of the other ww- and rr-BKPyV strains used in the study in Fig. S2 and their respective frequency is indicated.

**Table S1.** List of primers

| **Forward primers** | **Sequence (5'-3')** | **Amplicon length** |
| --- | --- | --- |
| BKTT7_UMI_NGS_F | TCTTTCCCTACACGACGCTCTTCCGATCTNNNNNNNNCCCTGTTAAGAACTTTATCCATTT | 518 |
| NCCR427_UMI_NGS_F | TCTTTCCCTACACGACGCTCTTCCGATCTNNNNNNNNCTTTATCCATTTTTGCAAAAATTG | 506 |
| NCCR413_UMI_NGS_F | TCTTTCCCTACACGACGCTCTTCCGATCTNNNNNNNNGCAAAAATTGCAAAAGAATAG | 492 |
| NCCR398_UMI_NGS_F | TCTTTCCCTACACGACGCTCTTCCGATCTNNNNNNNNGAATAGGGATTTCCCCAAATATTTTTG | 477 |
| NCCR384_UMI_NGS_F | TCTTTCCCTACACGACGCTCTTCCGATCTNNNNNNNNCCAAATATTTTTGCTAGGCC | 463 |
| NCCR371_UMI_NGS_F | TCTTTCCCTACACGACGCTCTTCCGATCTNNNNNNNNCTAGGCCTCAGAAAAAGCCTC | 450 |
| NCCR361_UMI_NGS_F | TCTTTCCCTACACGACGCTCTTCCGATCTNNNNNNNNGAAAAAGCCTCCACACCCTTAC | 440 |
| NCCR352_UMI_NGS_F | TCTTTCCCTACACGACGCTCTTCCGATCTNNNNNNNNCTCCACACCCTTACTACTTGAG | 432 |
| NCCR263_UMI_NGS_F | TCTTTCCCTACACGACGCTCTTCCGATCTNNNNNNNNGCTGCTAACCCATGGAATGTAG | 343 |
| NCCR178_UMI_NGS_F | TCTTTCCCTACACGACGCTCTTCCGATCTNNNNNNNNGAAACCCCGCCCCTAAAATTCTC | 258 |
| NCCR89_UMI_NGS_F | TCTTTCCCTACACGACGCTCTTCCGATCTNNNNNNNNCGAGCCTAGGAATCTTGGCCTTG | 169 |
|  |  |  |
| **Reverse primer** |  |  |
| BKTT8_UMI_NGS_R | GTGACTGGAGTTCAGACGTGTGCTCTTCCGATCTNNNNNNNNAACTTTCACTGAAGCTTGTCGT |  |

*Flow cytometry*

RPTEC were detached using TrypLE Express (Gibco), washed twice with PBS and incubated for 30 min at 4°C in fixation/permeabilization solution (BD Cytofix/Cytoperm Fixation/Permeabilization Kit, Cat. No. 554714). Cells were washed once in pre-cooled 1x BD Perm/Wash buffer (BD Cytofix/Cytoperm Fixation/Permeabilization Kit, Cat. No. 554714), before being resuspended in pre-cooled staining buffer (1x BD Perm/Wash buffer with 5% normal goat serum). Cells were subsequently stained with the primary antibody or an isotype control (Table S2). After washing with pre-cooled 1xBD Perm/Wash buffer, cells were stained with an Alexa Fluor 488-conjugated secondary antibody (Table S2). All antibodies were used at a concentration of 10 µg/ml diluted in staining buffer and incubated at 4°C for 60 min. After staining, cells were washed two times with pre-cooled 1xBD Perm/Wash buffer and fixed with PFA. All samples were analyzed with a FACS Canto II flow cytometer (BD Biosciences, Heidelberg, Germany) using the FACSDiva software (version 8.0.1).

**Table S2**. List of antibodies

| **Name** | **Supplier** | **Cat No.** | **Clone No.** | **Species** | **Application** |
| --- | --- | --- | --- | --- | --- |
| anti-CD13 | Abcam | ab7417 | WM15 | mouse monoclonal | FC |
| anti-cubilin | Abcam | ab244274 |  | rabbit polyclonal | FC |
| anti-megalin | RnD System | MAB9578-SP | 545606 | mouse monoclonal | FC |
| anti-NFκB p65 | Santa Cruz Biotechnology | sc-8008 X | F-6 | mouse monoclonal | IF/EMSA/IB |
| anti-NFκB p50 | Santa Cruz Biotechnology | sc-166588X | D-6 | mouse monoclonal | EMSA |
| anti-NFκB p52 | Santa Cruz Biotechnology | sc-7386X | C-5 | mouse monoclonal | EMSA |
| anti-Rel B | Santa Cruz Biotechnology | sc-48366X | D-4 | mouse monoclonal | EMSA |
| anti-c-Rel | Santa Cruz Biotechnology | sc-6955X | B-6 | mouse monoclonal | EMSA |
| anti-IKKγ | Cell Signaling Technology | 2685 |  | rabbit polyclonal | IB |
| anti-SV40 TAg | Santa Cruz Biotechnology | sc-53448 | Pab 416 | mouse monoclonal | IF/IB |
| anti-SV40 TAg | Cell Signaling Technology | 15729 | D1E9E | rabbit monoclonal | IF |
| anti-SV40 VP1 | Abcam | ab 53977 |  | rabbit polyclonal | IF/IB |
| anti-beta-actin | Sigma-Aldrich | A5441 | AC-15 | mouse monoclonal | IB |
| normal mouse IgG1 | Santa Cruz Biotechnology | sc-3877 |  | mouse polyclonal | FC/IF |
| rabbit IgG | Abcam | ab172730 | EPR25A | rabbit monoclonal | FC/IF |
| mouse IgM | Becton Dickinson | 557275 | C48-6 | mouse monoclonal | EMSA |
| Alexa Fluor 488 anti-mouse | Invitrogen | A11029 |  | goat polyclonal | FC/IF |
| Alexa Fluor 488 anti-rabbit | Invitrogen | A11034 |  | goat polyclonal | FC/IF |
| Alexa Fluor 546 anti-mouse | Invitrogen | A11030 |  | goat polyclonal | IF |
| Alexa Fluor 546 anti-rabbit | Invitrogen | A-11035 |  | goat polyclonal | IF |
| anti-mouse HRP | Sigma-Aldrich | A9044 |  | rabbit polyclonal | IB |
| anti-rabbit HRP | Sigma-Aldrich | A6154 |  | goat polyclonal | IB |

*^FC: flow cytometry, IF: immunofluorescence,^* *^EMSA: electrophoretic mobility shift assay, IB: immunoblot^*

*Immunoblot*

Immunoblot analysis of equal amounts of protein extracts were performed as previously described [3]. Primary antibodies directed against the viral proteins VP1 and TAg or against p65, IKKγ or beta actin (loading control) were used together with HRP-conjugated secondary antibodies (Table 1).

*Immunofluorescence*

RPTEC seeded on coverslips were fixed with 4% paraformaldehyde for 10 min, washed three times with PBS, permeabilized with PBS-0.2% triton X-100 for 5 min, washed three times with PBS and blocked for at least one hour with PBS - 0.1% Tween 20 (PBST) supplemented with 5% bovine serum albumin (BSA). Cells were incubated with the primary antibody (Table S2) diluted in PBST-1% BSA for one hour at room temperature. Cells were washed three times with PBST and incubated with Alexa-conjugated secondary antibodies (Table S2) for one hour. Samples were washed three times with PBST and the nuclei were counterstained with DAPI (200 ng/ml in methanol) for 2 min at room temperature. Pictures were taken with a Zeiss LSM900 confocal microscope, objective Plan-Apochromat 40x/1.3 Oil DIC (UV) VIS-IR M27 (Carl Zeiss, Oberkochen, Germany, Fig.S1b and 5a) or with a Leica DMI6000 B fluorescence microscope, objective HC PL FLUOTAR L 20x/0.40 DRY (Leica, Wetzlar, Germany, Fig. 2i and 4a).

For quantification of the number of TAg and VP1 positive cells, the fluorescence intensities in the nuclear region of each cell were analyzed with custom-written routines in Matlab (Mathworks, Natick, USA) as follows: Images of each color channel were first denoised with a 3x3 pixel median filter and then linearly deconvolved (Wiener filtered) with a Gaussian Point Spread Funktion of 250 nm FWHM. Binary masks covering all nuclei were generated by thresholding the resulting images in the DAPI channel. To avoid holes in these masks, „morphological closing“ was applied to the binary images with a disk of 2 pixels diameter. In the next step touching nuclei were separated: The binary mask was smoothed with a large (30 pixels FWHM) gaussian function, and watershedding was applied to separate the nuclei. Nuclei touching the image border were excluded from further analysis. The mean fluorescence intensity in the 488-channel and/or 546-channel in each nuclear region were then calculated based on the binary masks. For quantification of p65 nuclear translocation, the median intensity inside the nuclei was compared to the median intensity of the region immediately surrounding the nuclei.

*Neutral red uptake assays*

To determine cell viability by neutral red uptake assay, cells were incubated with 50 µg/ml of neutral red (Sigma-Aldrich) in BKRM for two hours at 37°C, washed twice with PBS and the dye was extracted using citrate buffer containing 50% ethanol, 30.6 mM disodium citrate and 19.4 mM HCl. Absorption of the supernatant was measured photometrically at 550 nm (reference at 405 nm).

*Transmission Electron Microscopy (TEM)*

BKPyV-infected RPTEC were fixed at 21 dpi with 2.5% glutaraldehyde in 0.05 M HEPES and stored at 4°C. RPTECs were processed for thin section TEM using a standard protocol as previously described [4]. Briefly, cells were scraped from the support, embedded in low-melting point agarose, fixed with osmium tetroxide and contrasted *en bloc* with tannic acid and uranyl acetate. Finally, samples were dehydrated in ethanol and embedded in epon resin. Ultrathin sections (60–70 nm) were cut with a diamond knife using a Leica UC7 ultramicrotome and stained with uranyl acetate and lead citrate. Sections were examined with a JEOL JEM-2100 TEM at 200 kV and a Tecnai Spirit TEM at 120 kV. Images were recorded with an EMSIS Veleta CCD camera (2048 by 2048 pixel) or a Thermo Fisher Scientific Eagle CCD camera (4096 by 4096 pixel).

*siRNA transfection*

RPTEC seeded in BKRM at 20,000 cells/well in a 96 well plate were transfected one day later with 1 pmol/well of siRNA directed against p65 (ON-TARGETplus Human RELA SMARTpool siRNA, ref. L-003533-00-0005, Dharmacon) or IKK-γ (ON-TARGETplus Human IKBKG siRNA, pool of 4 siRNAs , ref. LQ-003767-00-0002, Dharmacon), or of the control siRNA (ON-TARGETplus Non-targeting Control siRNA #2, Dharmacon), and 0.3 µl of Lipofectamine RNAimax (Thermo Fisher Scientific) in 70 µl/well (siRNA final concentration of 14.3 nM) according to the manufacturer’s instructions. Four days later, the cells were infected with BKPyV (MOI 0.1) as described earlier. Seven days post infection (eleven days post transfection), BKPyV load in the supernatant was determined by qPCR and cell growth/viability was determined by neutral red assay. To control the silencing efficiency, RPTEC seeded in 12 well plates (200,000 cells/well) were transfected with 10 pmol/well of siRNA and 3 µl of Lipofectamine RNAimax in 700µl/well. Cells were harvested 11 days post transfection for immunoblot analysis.

*Electrophoretic mobility shift assay (EMSA)*

Double-stranded DNA oligonucleotides (BK1: 5’-AAGAATAGGGATTTCCCCAAATA-3’, BK1 mut: 5’-AAGAATAGGGATTTGACCAAATA-3’, pBK2W: 5’-TCAGGAAGGAAAGTGCATGACTGGG-3’, pBK2D1: 5’-TCAGGAAGGAAAGTGCATGACTCAC-3’, pBK2D2: 5’-TCAGGAAGGAAAGTGCATGACAGAC-3’, pBK3D: 5’-ACTCACAGGGGAATGCAGCCAAAC-3’ and the NF-κB BS sequence from the mouse κ light chain enhancer [5]: 5’-AGCTTCAGAGGGGACTTTCCGAGAGG-3’) were labeled with (γ-^32^P)-ATP (Hartmann Analytic, Braunschweig, Germany) using T4 polynucleotide kinase (New England Biolabs, Ipswich, MA). Nuclear extracts (NE) of uninfected and infected (either WWM12 or Dunlop with a MOI of 0.5) RPTEC either unstimulated or stimulated with 1000 U/ml TNF-α for 30 min at 37°C were prepared 7 dpi according to [6]. Roughly, cells were harvested in PBS and centrifuged for 5 min at 960 x g at 4°C. The cell pellet was resuspended in 400 µl buffer A (10 mM Hepes [pH 7.9], 10 mM KCl, 0.1 mM EDTA [pH 8.0], 0.1 mM EGTA, 1 mM DTT, 0.6 mM PMSF, 10 µg/ml aprotinin and 10 µg/ml leupeptin), vortexed for 10 sec, and incubated on ice for 15 min. After incubation, 50 µl NP-40 were added to the cell mixture and vortexed for 10 sec, followed by centrifugation for 1 min at 15,000 x g at 4°C. The pellet was then resuspended in 20-50 µl buffer C (20 mM Hepes [pH 7.9], 0.4 mM NaCl, 1 mM EDTA [pH 8.0], 1 mM EGTA, 1 mM DTT, 0.6 mM PMSF, 10 µg/ml aprotinin and 10 µg/ml leupeptin) and incubated overnight on a shaker at 1,400 rpm at 4°C. The suspension was then centrifuged for 5 min at 15,000 x g at 4°C and the supernatant was stored at -80°C. The protein concentration was measured using Bradford assay. The NFκB binding reaction was performed in a total volume of 20 µl binding buffer (2 µl Ten50 (Tris-HCl [pH 7.5], 50 mM NaCl, 1 mM EDTA [pH 8.0]), 8 µl reaction buffer (2 µl of 10x binding buffer (100 mM Tris-HCl [pH 7.5], 10 mM EDTA [pH 8.0], 500 mM NaCl, 50% glycerol), 2 µl of buffer D+ (20 mM Hepes, 20% glycerol, 100 mM KCl, 0.05 mM EDTA [pH 8.0], 1% NP-40), 2 mg/ml poly dI-dC, 20 mg/ml BSA, 0.3 mg/ml aprotinin, 4 mM PMSF, 25 mM DTT), as previously described [7, 8] using 5 µg of NE and 250 pg of labeled double-stranded oligonucleotides. In supershift experiments, the NE were pre-incubated on ice for 20 min with 2 µg monoclonal antibodies (Santa Cruz Biotechnology) against NF-κB p65 (F-6 X), p50 (D-6 X), p52 (C-5 X), RelB (D-4 X), cRel (B-6 X) or isotype control (purified mouse IgM, Becton Dickinson) before adding the probe. The protein-DNA complexes were separated in a non-denaturing 4% polyacrylamide gel by electrophoresis (1.5 h at 180V). The gel was then fixed in buffer containing 10% ethanol (v/v) and 10% glacial acetic acid (v/v) for 45 min at room temperature, followed by a 2 h air drying step on Whatman paper before exposure on a photographic film.

**Supplementary Figures**


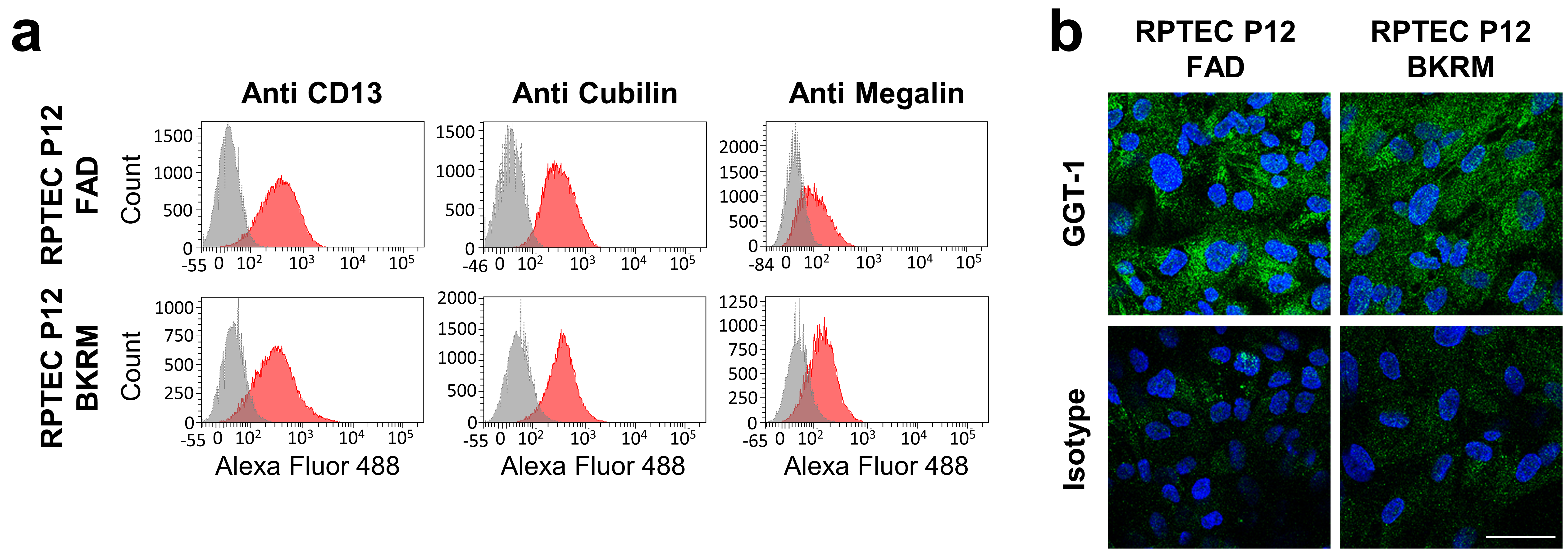


**Fig. S1** Expression of kidney proximal tubule markers in RPTEC cultured in FAD and after switch to BKRM. Flow cytometry (**a**) and immunofluorescence (**b**) analysis of kidney proximal tubule markers CD13, cubilin, megalin (**a**) (in red, isotype control in grey) and GGT-1 (**b**) (in green, DAPI in blue) in RPTEC after 12 passages (P12) in FAD culture medium or after switching from FAD to BKRM at P12 for 10 days. Representative experiments out of at least three independent replicates are shown. (**b**) Scale bar = 50 µm





**Fig. S2** (**a**) NCCR sequence alignment of the archetype WWM12, WWT, WWM5, of the representative rearranged sequences of M401 and of the rearranged Dunlop BKPyV strains. The different blocks of the NCCR are indicated by a color code. Putative BS for TNF-α-activated transcription factors predicted by PROMO 3.0 [9, 10] are indicated, as well as the sequence of oligonucleotides used in EMSA (**b**) Frequency of M401 representative rearranged NCCR sequences.





**Fig. S3. Analysis of point mutations, deletions and insertions in inocula and 10 dpi samples (BKRM) of ww-strains shown in Fig. 1 and Table 1.** The percentage of sequences harboring mutations, deletions or insertions at the indicated positions compared to the respective archetype NCCR (WWM12, WWT, or WWM5) for inocula and 10 dpi samples (BKRM) is represented in blue and orange bars respectively. The positions in archetype NCCRs are indicated on the x-axis, as well as the position of the O (black), P (green), Q (yellow), R (grey) and S (red) blocks. The star (*) indicates frequent deletions or insertions of 1 nucleotide at position 131 and 132 respectively, which are the first bases of a stretch of 9 consecutive adenines and may therefore result from amplification or sequencing artifacts.


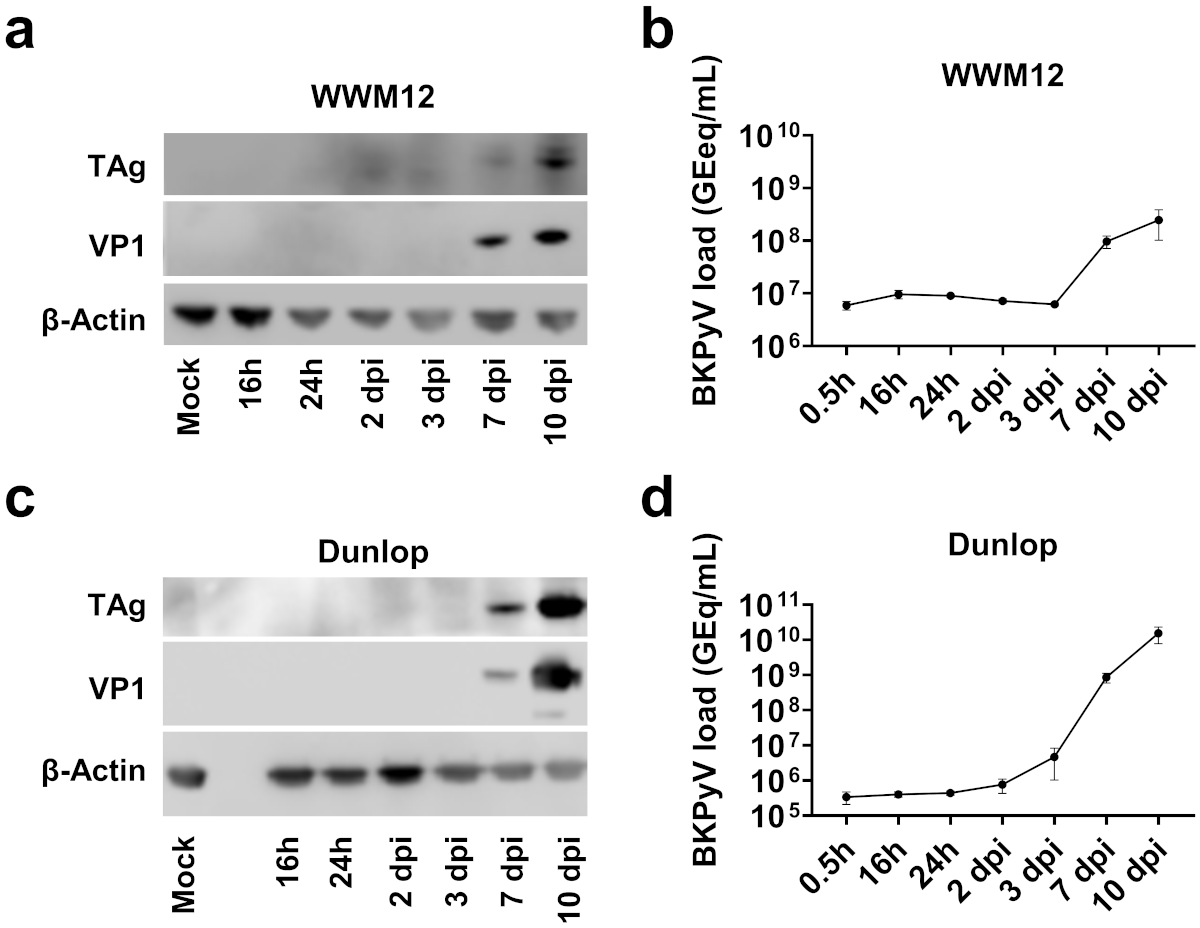


**Fig. S4.** Analysis of ww- and rr-BKPyV replication at early time points. RPTEC seeded in BKRM were infected with WWM12 or Dunlop BKPyV (MOI 0.1). (**a, c**) TAg and VP1 expression was analyzed in infected RPTEC at the indicated dpi by immunoblot (loading control: β-actin). (**b, d**) Viral replication was analyzed in parallel by qPCR on supernatants (Mean±SEM of at least 3 independent experiments).


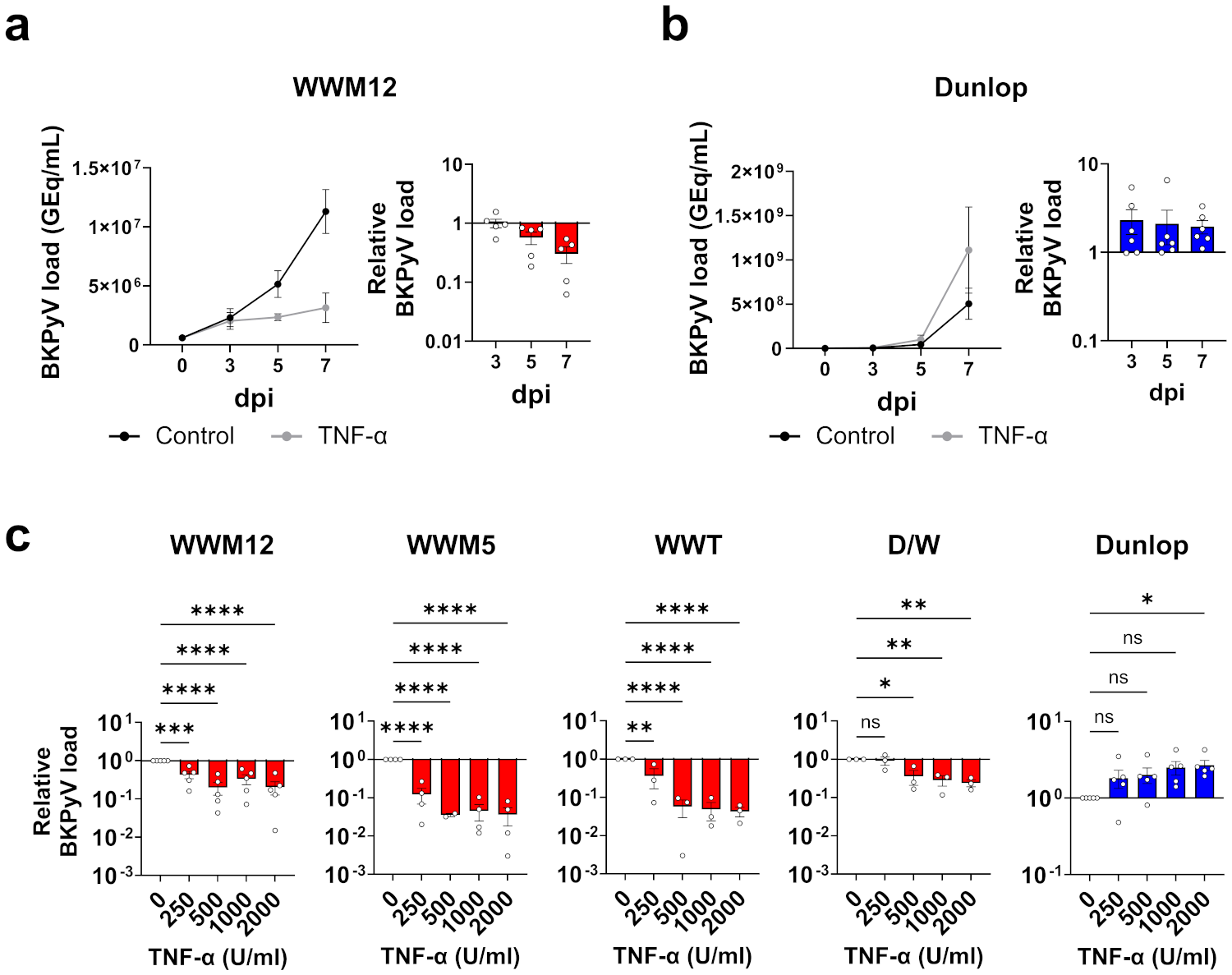


**Fig. S5** Effect of TNF-α on archetype and rearranged BKPyV replication at early time points or using different TNF-α concentrations. (**a-c**) RPTEC seeded in BKRM were infected with the indicated strains at MOI 0.1 (**a**, **b**), or MOI 0.02 (**c**, all strains except D/W at MOI 0.05) and treated with 1000 U/ml of TNF-α (**a, b**), or with the indicated TNF-α concentrations (**c**). Viral replication was analyzed by qPCR on viral supernatants at the indicated time points (**a, b**) or at 7 dpi (**c**). The mean ± SEM of viral load in genome equivalent per milliliter (GEq/mL) is shown in **a** and **b** (left panels). The mean ± SEM of relative viral load in the TNF-α-treated conditions normalized to the respective unstimulated controls of at least 3 independent experiments in technical triplicates is shown in **a, b** (right panels) and in **c**. (Statistics: (**c**) one-way ANOVA with Dunnett’s multiple comparisons test).


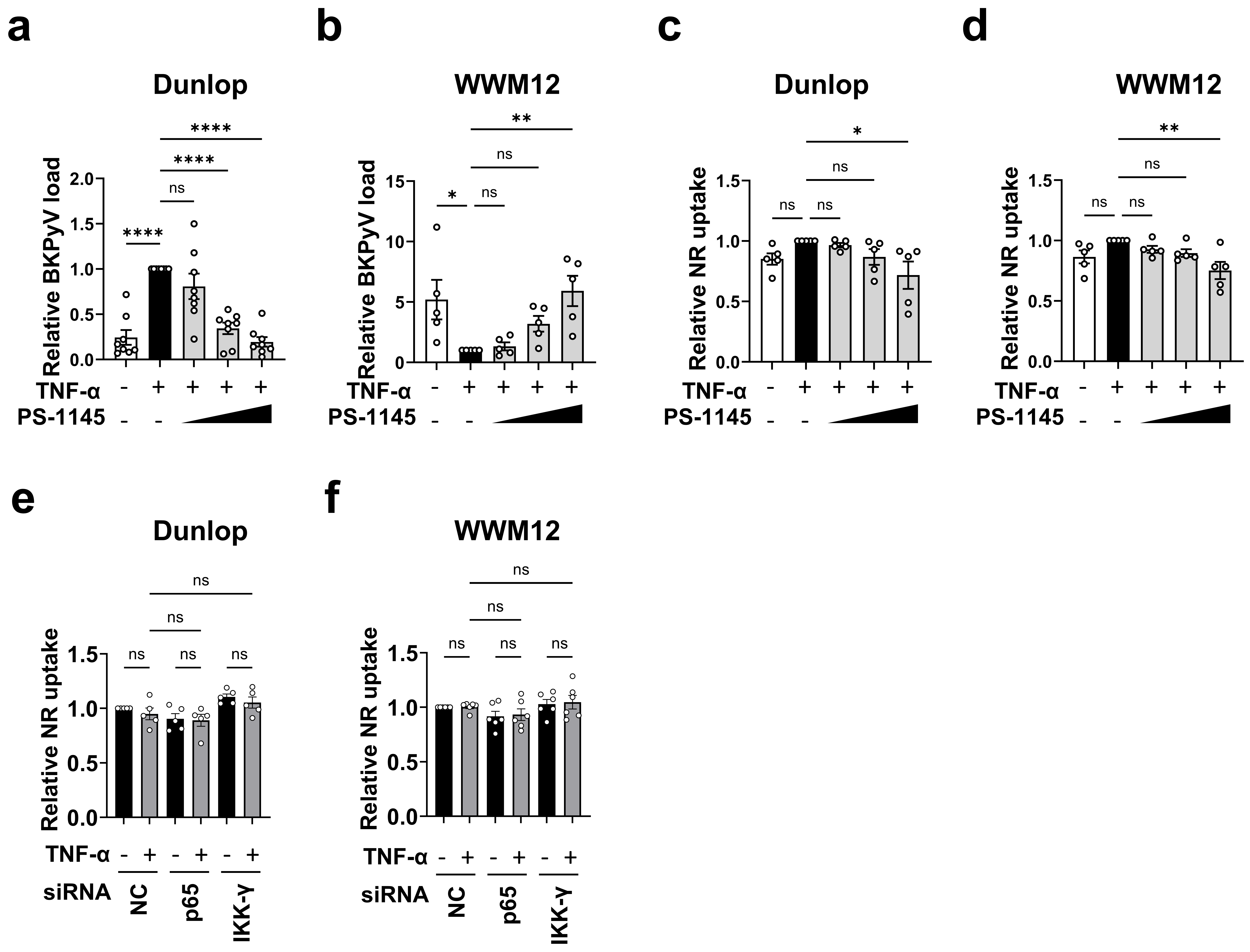


**Fig. S6** (**a-d**) Effect of PS-1145 on rr- and ww-BKPyV response to TNF-α. RPTEC were infected with Dunlop or WWM12 (MOI 0.02) in presence or not of TNF-α (1000 U/ml) and increasing concentrations of PS-1145 (1, 5 or 10 µM). DMSO was used as a negative control. (**a, b**) Viral replication was analyzed 7 dpi by qPCR on viral supernatants (**c, d**). RPTEC growth/viability was assessed by neutral red assay in parallel with the PS-1145 experiments (**c, d**) or with the siRNA experiments show in Fig 5c and d (**e, f**). The mean ± SEM of relative values normalized to the “+ TNF-α, no PS-1145” condition (**a-d**) or to the “unstimulated NC siRNA” condition (**e, f**), of at least 3 independent experiments in technical triplicates is shown. (Statistics: one-way ANOVA with Dunnett’s multiple comparisons test).


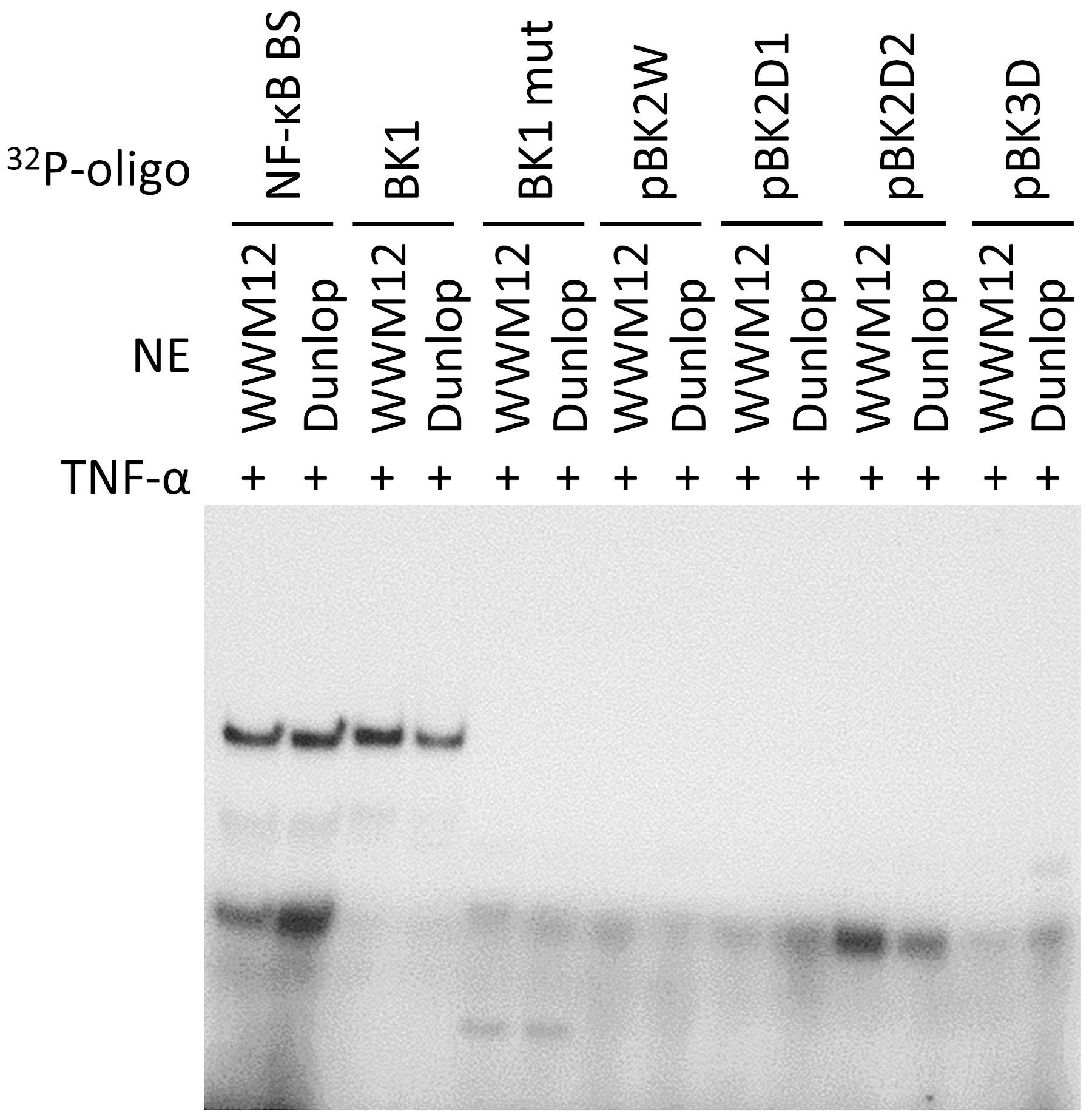


**Fig. S7** Binding activity of TNF-α -stimulated BKPyV-infected RPTEC nuclear extracts to the putative NF-κB BS in WWM12 and Dunlop NCCR. An EMSA was performed using ^32^P-labelled double-stranded DNA oligonucleotides containing the NF-κB BS from the immunoglobulin κ light chain enhancer (NF-κB BS), the putative NF-κB BS predicted in BKPyV WWM12 or Dunlop NCCR (BK1, pBK2W, pBK2D1, pBK2D2, pBK3D) incubated with nuclear extract (NE) of WWM12- or Dunlop-infected RPTEC (MOI 0.5) stimulated with 1000 U/ml of TNF-α for 30 min.

Supplementary References

1. Bryksin, A.V., Matsumura, I.: Overlap extension PCR cloning: a simple and reliable way to create recombinant plasmids. BioTechniques **48**(6), 463–465 (2010). doi: 10.2144/000113418

2. Gosert, R., Rinaldo, C.H., Funk, G.A., Egli, A., Ramos, E., Drachenberg, C.B., Hirsch, H.H.: Polyomavirus BK with rearranged noncoding control region emerge in vivo in renal transplant patients and increase viral replication and cytopathology. The Journal of experimental medicine **205**(4), 841–852 (2008). doi: 10.1084/jem.20072097

3. Marthaler, A.M., Podgorska, M., Feld, P., Fingerle, A., Knerr-Rupp, K., Grässer, F., Smola, H., Roemer, K., Ebert, E., Kim, Y.-J., Bohle, R.M., Müller, C.S.L., Reichrath, J., Vogt, T., Malejczyk, M., Majewski, S., Smola, S.: Identification of C/EBPα as a novel target of the HPV8 E6 protein regulating miR-203 in human keratinocytes. PLoS pathogens **13**(6), e1006406 (2017). doi: 10.1371/journal.ppat.1006406

4. Laue, M.: Electron microscopy of viruses. Methods in cell biology **96**, 1–20 (2010). doi: 10.1016/S0091-679X(10)96001-9

5. Sen, R., Baltimore, D.: Inducibility of kappa immunoglobulin enhancer-binding protein Nf-kappa B by a posttranslational mechanism. Cell 47(6), 921–928 (1986). doi: 10.1016/0092-8674(86)90807-x

6. Schreiber, E., Matthias, P., Müller, M.M., Schaffner, W.: Rapid detection of octamer binding proteins with 'mini-extracts', prepared from a small number of cells. Nucleic acids research 17(15), 6419 (1989). doi: 10.1093/nar/17.15.6419

7. Hess, S., Rensing-Ehl, A., Schwabe, R., Bufler, P., Engelmann, H.: CD40 function in nonhematopoietic cells. Nuclear factor kappa B mobilization and induction of IL-6 production. Journal of immunology (Baltimore, Md. : 1950) 155(10), 4588–4595 (1995)

8. Haglund, R.E., Rothblum, L.I.: Isolation, fractionation and reconstitution of a nuclear extract capable of transcribing ribosomal DNA. Molecular and cellular biochemistry 73(1), 11–20 (1987). doi: 10.1007/BF00229371

9. Farré, D., Roset, R., Huerta, M., Adsuara, J.E., Roselló, L., Albà, M.M., Messeguer, X.: Identification of patterns in biological sequences at the ALGGEN server: PROMO and MALGEN. Nucleic acids research 31(13), 3651–3653 (2003). doi: 10.1093/nar/gkg605

10. Messeguer, X., Escudero, R., Farré, D., Núñez, O., Martínez, J., Albà, M.M.: PROMO: detection of known transcription regulatory elements using species-tailored searches. Bioinformatics 18(2), 333–334 (2002). doi: 10.1093/bioinformatics/18.2.333
